# Tea Polyphenol Epigallocatechin Gallate and the Gut–Health Axis: Unraveling Structural Characteristics, Metabolic Pathways, and Systemic Benefits

**DOI:** 10.1016/j.advnut.2025.100545

**Published:** 2025-10-15

**Authors:** Jiaying Yang, Wei Chen, Jiayi Chen, Dengchao Xie, Yuefei Wang, Jihong Zhou

**Affiliations:** Department of Tea Science, College of Agriculture and Biotechnology, Zhejiang University, Hangzhou, China

**Keywords:** tea polyphenols, gut microbiota, biological activities, regulatory mechanisms, health benefits

## Abstract

Dietary components significantly impact human health, influencing diverse physiological processes from metabolic homeostasis to cognitive function and aging. Tea, a widely consumed functional beverage rich in antioxidants, has gained attention for its health benefits. Epigallocatechin gallate (EGCG), the most abundant and bioactive catechin in green tea, is renowned for its potent biological activities. However, the direct absorption of EGCG is limited due to its low oral bioavailability, with a substantial portion reaching the colon where it interacts extensively with gut microbiota. This microbial interplay is crucial for EGCG’s biotransformation and the realization of its health-promoting potential, yet the underlying mechanisms remain to be fully elucidated. This review synthesizes EGCG's structural features, metabolism, and interactions with gut microbiota, summarizing its roles in gut health and systemic effects through gut-related axes, and outlines future research. First, it elaborates EGCG's structural features, as a flavan-3-ol with a polyphenolic structure containing multiple hydroxyl groups, whose antioxidant and bioactive properties are associated with the specific arrangement of benzene rings and the gallate moiety. Second, it outlines its metabolic process, limited absorption in the small intestine, enzymatic metabolism in the small intestine and liver (including methylation, glucuronidation, and sulfation), and extensive biotransformation in the colon by gut microbiota into metabolites such as epigallocatechin and gallic acid. Third, it explores its effects on the gut, modulating gut microbiota composition by promoting beneficial bacteria and inhibiting pathogenic strains, enhancing intestinal barrier function by upregulating tight junction proteins, and promoting the production of short-chain fatty acids. Finally, it elucidates how EGCG modulates key gut-related health pathways and its broader implications for systemic health through various interconnected gut axes, including the gut–liver, gut–brain, gut–renal, and gut–lung axes, and concludes by outlining prospective research directions aimed at further elucidating the potential of EGCG in promoting health.

## Graphical abstract

**Figure:**
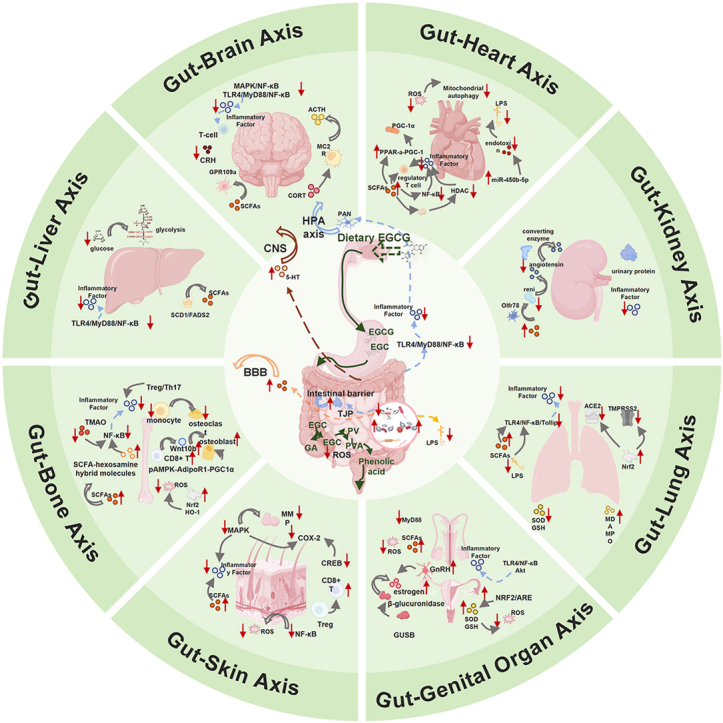

**Statements of significance**

This review critically synthesizes the pivotal role of the gut microbiota in mediating the significant health benefits of epigallocatechin gallate (EGCG), the primary bioactive polyphenol in tea. By integrating EGCG’s structure, metabolism, and gut–microbe interactions, this work positions the gut as the critical nexus for EGCG’s action and highlights its promise as a dietary strategy for systemic health promotion.

## Introduction

Diet plays a crucial role in human health, influencing daily activities, cognition, exercise, and aging [[1], [2], [3]]. With growing awareness of its importance, dietary therapy has expanded from a tool for managing diabetes to a foundation of national nutrition programs. Tea is gaining popularity as a functional beverage, valued for its rich content of antioxidants and essential vitamins, which contributes to a wide range of health benefits [4,5]. It is estimated that 3.5 billion cups of tea are consumed worldwide every day [6] and global tea consumption is expected to grow at a compound annual growth rate of 6.13% by 2027 [7]. The health benefits of tea stem from its bioactive compounds, including antioxidants, vitamins, carbohydrates, proteins, minerals, chlorophyll, and polyphenols [8]. However, the most representative bioactive compounds, tea polyphenols, which account for 18%–36% of the dry weight of tea leaves are poorly absorbed in human intestine and instead rely on interactions with the gut microbiota to exert their health effects [9,10].

Among tea polyphenols, epigallocatechin gallate (EGCG) stands out as the most abundant and biologically active compound and accounts for ∼50% of the total polyphenol content in green tea [8,11], significantly influencing human health regulation [12]. During digestion, only a small portion of the ingested EGCG can enter the bloodstream, thereby most EGCG and its metabolites bypass direct absorption, reaching the colon where they engage with the various gut microbiota. Here, EGCG is degraded into smaller compounds, such as epigallocatechin (EGC) and gallic acid (GA), or transformed through processes like methylation, glucuronidation, sulfation and ring fission biotransformation, which accumulate in the body to drive its bioactivity [8,13]. Beyond metabolism, EGCG and its metabolites promote intestinal health by enhancing the diversity of beneficial bacteria, strengthening the intestinal barrier, and optimizing nutrient metabolism [14,15]. In recent years, the promoting effect of EGCG on intestinal health has been discussed in numerous in vitro and in vivo studies. For example, Liu et al. [16], Li et al. [17], Liu et al. [18], and Xie et al. [19] demonstrated that EGCG alleviated intestinal injury by regulating fatty acid metabolism, modulating the microbiota-tryptophan-aryl hydrocarbon receptor pathway (where gut microbes convert dietary tryptophan into indole derivatives that activate aryl hydrocarbon receptor (AhR, a ligand-activated transcription factor crucial for immune regulation and maintaining barrier function) to sustain mucosal IL-22 production and epithelial barrier integrity), inhibiting the Toll-like receptor-4 (TLR4)-nuclear factor kappa B (NF-κB)/ mitogen-activated protein kinase (MAPKs)-NOD-like receptor family pyrin domain containing 3 (NLRP3) inflammatory cascade reaction and regulating nuclear factor erythroid 2-related factor 2 (Nrf2) signaling pathway.

Gut microbiota as a key player in maintaining overall health, with dysbiosis linked to not only gastrointestinal issues but also been implicated in the development of chronic conditions such as obesity, colitis and neurodegenerative diseases [20,21]. Therefore, dietary interventions targeting gut health have emerged as a promising approach to prevent and manage these conditions. A growing body of evidence supports the efficacy of such interventions, demonstrating improvements in gut microbiota composition and corresponding health outcomes after the consumption of dietary EGCG [22]. Specifically, EGCG can alleviate corresponding chronic diseases (including cardiovascular diseases, neurodegenerative diseases and metabolic diseases) by upregulating the abundance of beneficial bacteria, inhibiting the abundance of pathogenic bacteria and altering microbial metabolites through the gut–health axis pathway [[23], [24], [25]]. Therefore, conducting a systematic review and in-depth discussion on the potential of EGCG to promote systemic benefits through the gut–health axis is of great scientific significance, and it also has positive implications for improving public health and promoting disease prevention efforts.

This review provides a detailed overview of EGCG's structural characteristics, absorption, and metabolism with a special emphasis on its interactions with the gut microbiota and their health implications. It delves into the mechanisms by which EGCG modulates critical gut-related health pathways and concludes by outlining future research directions.

## Structural Characteristics and Intestinal Metabolism of EGCG

### Chemical structure of EGCG

**FIGURE 1:**
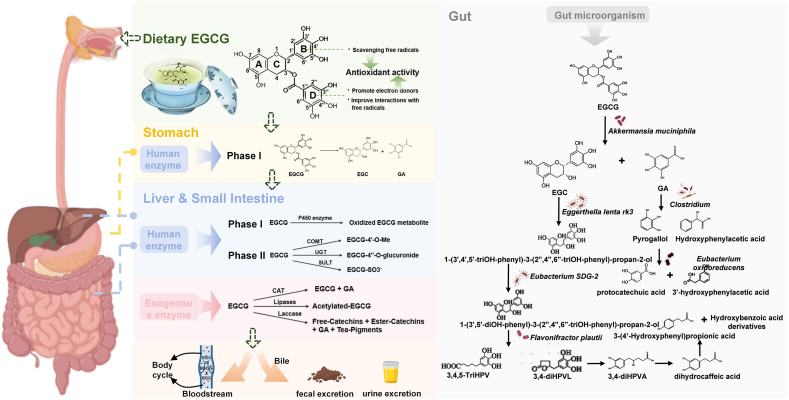
The metabolic pathways of EGCG in the gastrointestinal tract and liver: (A) In the stomach, EGCG is hydrolyzed by gastric esterases into epigallocatechin (EGC) and gallic acid (GA), marking the initiation of phase I metabolism. (B) In the liver and small intestine, cytochrome P450 (CYP) enzymes oxidize EGCG to enhance its polarity, followed by phase II conjugation reactions. Methylation (via catechol-O-methyltransferase, COMT), glucuronidation (via UDP-glucuronosyltransferases, UGTs), and sulfation (via sulfotransferases, SULTs) improve water solubility, facilitating excretion via urine or bile. and (C) In the colon, gut microbes further metabolize GA and EGC. Flavonifractor plautii converts GA to pyrogallol (PG), whereas Adlercreutzia equolifaciens transforms GA to hydroxyphenylacetic acid (HPA). Eggerthella lenta and Eubacterium species cleave EGC’s C-ring to form trihydroxybinol (TriHB). CAT, catalase; EGCG, epigallocatechin gallate; P450, cytochrome P450; UDT, uridine diphosphate.

EGCG is a flavan-3-ol within the catechin group of polyphenolic compounds prevalent in green tea. Its chemical structure underlies its diverse biological activities, particularly its potent antioxidant effects. The molecule is composed of 2 benzene rings (A and B) and a dihydropyran heterocycle (C-ring) linked by a 3-carbon chain [[26], [27], [28]]. As shown in Figure 1, EGCG’s antioxidant efficacy stems from its 8 hydroxyl groups positioned at key sites. In particular, the 3′,4′,5′-trihydroxyphenyl (pyrogallol) structure on the B-ring is critical for free radical scavenging through efficient electron donation from its hydroxyl groups [27], whereas the gallate group at the C-3 position further enhances antioxidant capacity through efficient electron donation, which aligns with EGCG’s structural advantage of 8 free hydroxyl groups as superior electron donors. These structural elements confer EGCG with a higher antioxidant efficacy compared with other catechins, such as epicatechin (EC) and EGC [28,29]. Moreover, EGCG predominantly exists as a cis-isomer, a configuration that positions the B-ring’s 3′,4′,5′-trihydroxy (pyrogallol) group and the C-ring in close spatial proximity. This specific 3-dimensional arrangement facilitates intramolecular hydrogen bonding and electron delocalization across the molecule, stabilizing the phenoxyl radical formed after electron donation and thereby enhancing the B-ring's ability to scavenge free radicals [28]. Notably, EGCG metabolites retain antioxidant activity via the preserved 3′,4′-dihydroxyphenyl structure, a feature also critical to small-molecule metabolites of aromatic phenolic acids [27].

### Bioavailability of EGCG

Despite its promising bioactivity, EGCG’s therapeutic potential is limited by its low bioavailability [27]. This is largely attributed to its instability under physiological conditions, poor absorption within the gastrointestinal tract, and restricted permeability across cellular membranes [8,27,30,31].

#### Stability in the digestive system

EGCG’s stability varies with pH, ionic strength, and temperature. In the stomach’s acidic environment (pH 1.5), it exhibits an oil–water partition coefficient (lg P) between –1 and 2, indicating favorable hydrophilic and lipophilic properties facilitating its transport across the gastric mucosa to the intestine [32,33]. However, in the intestine’s neutral to alkaline conditions (pH >7.8), its lg P drops below –1, rendering it highly hydrophilic and prone to autoxidation, which produces oxidative byproducts and impairs transmembrane transport [33,34]. In addition, the phenolic hydroxyl group on the B ring of EGCG is prone to auto-oxidation under neutral and alkaline conditions, which makes it easy to degrade in alkaline plasma and bile, thereby affecting its absorption and metabolism [35]. Metal ions like Cu^2+^ and Fe^2+^ accelerate oxidative degradation [36], whereas temperatures above 50°C experienced in external conditions, such as during tea brewing or food processing, trigger epimerization to (–)-gallocatechin gallate [28], further hastening degradation [26,34].

#### Intestinal absorption

As shown in Figure 1, EGCG’s high molecular weight and polar nature restricts its intestinal uptake, which occurs mainly through passive transcellular and paracellular diffusion, which is inherently inefficient [37]. Consequently, <1% of orally ingested EGCG is absorbed into systemic circulation [38]. For example, studies in mice indicate that only 0.16% of digested tea polyphenols enter the bloodstream, with most excreted in the urine and feces [39]. EGCG is prone to be metabolized by the microorganisms in the small intestine and large intestine [40]. Additionally, the absence of specific active transporters, combined with the activity of efflux proteins such as P-glycoprotein and multidrug resistance proteins on the intestinal epithelium, exacerbate this by pumping EGCG back into the intestinal lumen, further reducing its bioavailability [34,41].

#### Influence of food matrix

The food matrix also plays a critical role in modulating the bioavailability of EGCG. Dietary components such as proteins, fibers, and divalent metals can interact with EGCG, forming complexes that reduce its solubility and hinder absorption [11]. For example, casein has been shown to bind with EGCG, delaying its release and absorption in the intestine [42,43]. Similarly, interactions with dietary fibers and metals, such as calcium and magnesium, can decrease EGCG’s bioaccessibility [44,45]. Additionally, the gut microbiota can modulate the release of EGCG from the food matrix, either enhancing or inhibiting its absorption depending on the microbial composition [45].

### Metabolism of EGCG

EGCG undergoes extensive metabolism in the liver, small intestine, and gut, involving enzymatic and microbial processes that shape its bioactivity and excretion [39].

#### Enzymatic metabolism

EGCG is primarily metabolized in the stomach, liver, and small intestine through phase I and phase II reactions (Figure 1). In the stomach, gastric esterases initiate phase I metabolism by hydrolyzing EGCG’s ester bonds, releasing GA and EGC [26,27,46]. This step increases EGCG’s polarity and water solubility, preparing it for further metabolism. In the liver and small intestine, phase I metabolism continues via cytochrome P450-mediated oxidation, which introduces hydroxyl groups or modifies aromatic rings to enhance EGCG’s polarity. Subsequently, in phase II metabolism, EGCG is conjugated through methylation, glucuronidation, and sulfation. Methylation, primarily by catechol-O-methyltransferase (COMT), adds a methyl group to EGCG's C-4′ hydroxyl group, forming a more lipophilic compound for easier transport and distribution in the body [26,27,46]. Glucuronidation catalyzed by uridine diphosphate (UDP)-glucuronosyltransferase (UGT) especially subtypes UGT1A1, UGT1A8, and UGT1A9, produces EGCG glucuronides like EGCG-4"-O-glucuronide, which makes it more water-soluble and can be excreted via urine or bile [27]. Sulfation mediated by sulfotransferases (SULT), transferring a sulfate group to EGCG, to enhance its solubility and facilitating excretion [27,46]. After entering the liver through the portal vein, EGCG further undergoes sulfation, methylation, and glucuronidation to form various conjugates such as EGCG-4′-O-glucuronic acid [47]. Metabolic products can be secreted into bile and enter the gut–liver circulation. Unabsorbed EGCG enters the large intestine, where it is hydrolyzed by intestinal microorganisms into EGC and GA, and further cleaved into valerolactone metabolites [such as 5-(3′,4′,5′-trihydroxyphenyl)-γ-valerolactone, that is, M4 or EGC-M5] [48]. Ultimately, EGCG and its metabolites enter the systemic circulation to exert biological activity or are excreted from the body with feces or urine. In addition to human enzymes, exogenous enzymes also influence EGCG metabolism with tannase [catalase (CAT)] hydrolyzing EGCG into EGC and GA, lipases enhancing EGCG stability and antioxidant properties via acetylation, and laccase oxidizing EGCG into various bioactive compounds such as free catechins and tea pigments [27].

#### Microbial metabolism

Beyond enzymatic catalysis, gut microbiota plays a pivotal role in the metabolism of EGCG. Specifically, it mediates biological transformation of EGCG through enzymatic reactions such as hydrolysis, cyclization, dehydroxylation, endoesterification, and methylation [[49], [50], [51], [52]]. In a vitro study indicated that the process begins with the hydrolysis of EGCG into EGC and GA, followed by further microbial conversion of these compounds into smaller, bioactive metabolites, mainly catalyzed by *Akkermansia muciniphila*, a bacterium found in the mucus layer of the human gut [53]. Additionally, *Enterobacter aerogenes*, *Raoultella planticola*, *Bifidobacterium longum*, and *Klebsiella pneumoniae* also have been proven to be involved in this hydrolysis process [54]. Once GA is released, it is further metabolized by gut microbes into phenolic intermediates. For example, GA undergoes decarboxylation to form pyrogallol (benzene-1,2,3-triol), which is subsequently converted into smaller phenolic acids such as 3,4-dihydroxybenzoic acid (protocatechuic acid) and 3′-hydroxyphenylacetic acid through sequential dehydroxylation and oxidation [55]. These reactions involve a consortium of bacteria, including *Clostridium* and *Eubacterium oxidoreducens* [52,56,57]. EGC, the other hydrolysis product, undergoes more complex transformations. Its C-ring is reduced and cleaved primarily by *Eggerthella lenta rk3*, producing intermediates such as 1-(3′,4′,5′,-trihydroxyphenyl)-3-(2′′,4′′,6′′,-trihydroxyphenyl)-propan-2-ol (3′,4′,5′-triH-2′′,4′′,6′′-triHPP-2-ol) [58]. This intermediate is further modified by regioselective dehydroxylation (preferentially at the 4′-position) mediated by *E. strain SDG-2*, generating 1-(3′,5′-dihydroxyphenyl)-3-(2′′,4′′,6′′-trihydroxyphenyl)-propan-2-ol (3′,5′-diH-2′′,4′′,6′′-triHPP-2-ol), which step is strictly dependent on the (3R)-configuration of the flavan-3-ol substrate [52]. Subsequently, these diphenylpropan-2-ol intermediates are further processed by *Flavonifractor plautii*, a bacterium also found in human gut, which catalyzes A-ring fission followed by lactonization to form 5-(3′,4′,5′-trihydroxyphenyl)-γ-valerolactone (3,4-diHPVL) and 5-(3′,4′,5′-trihydroxyphenyl) valeric acid (3,4,5-TriHPV) [[58], [59], [60], [61]]. 3,4-diHPVL undergoes lactone ring opening via bacterial esterases, generating 4-hydroxy-5-(3′,4′-dihydroxyphenyl)valeric acid (3,4-diHPVA), which further undergoes β-oxidation and decarboxylation reactions that shorten the side chain, producing 3-(3′,4′-dihydroxyphenyl)propionic acid (dihydrocaffeic acid), 3-(4′-hydroxyphenyl)propionic acid and finally hydroxybenzoic acid derivatives [52,56,60]. Notably, the metabolism of EGCG-derived intermediates exhibits high interindividual variability due to differences in gut microbiota composition. For example, strains such as *Clostridium orbiscindens* and *Butyrivibrio* contribute to the diversity of metabolite profiles, particularly in the production of methylated derivatives like homovanillic acid (4-hydroxy-3-methoxyphenylacetic acid) [56]. These metabolites including PVL conjugates (PVL-3′-glucuronide and PVL-4′-sulfate), absorbed by the colon, enter the bloodstream via the portal vein or are excreted in feces [58,59]. The metabolism of EGCG in the gut microbiota enhances its bioavailability and produces bioactive metabolites with antioxidant [62], anti-inflammatory [63], and anticancer [64,65] effects. The microbial diversity in the gut is a key determinant of the extent and efficiency of EGCG metabolism, with certain bacteria like *A. muciniphila, E. lenta*, and *F. plautii* playing pivotal roles in transforming EGCG into absorbable compounds for systemic circulation and beneficial effects.

### Strategies to enhance EGCG bioavailability and systemic delivery

The limited systemic delivery of EGCG is largely attributed to its poor stability and low bioavailability. Oral EGCG is highly susceptible to degradation in the gastrointestinal environment, with only a small fraction being absorbed into the bloodstream and the instability of EGCG under intestinal and blood conditions leads to rapid degradation, reducing its effective concentration for exerting biological activities [31]. Moreover, the lack of specific receptors for EGCG on intestinal epithelial cells (IECs) and the presence of efflux proteins further hinder its absorption [31]. To address these challenges, recent formulation innovations have been explicitly engineered to safeguard the molecule throughout gastric transit and to release it in a controlled fashion within the intestinal lumen [[66], [67], [68]].

Recent investigations converge on 3 technological directions. Cyclodextrin–protein ternary nanocomplexes, exemplified by the EGCG-casein-sulfobutylether-β-cyclodextrin ternary composite nanoparticles in Li et al.’s [69] research, achieve encapsulation efficiencies exceeding 95% and enhance the stability of EGCG in the gastrointestinal environment while enabling sustained release under simulated gastrointestinal conditions. Ultrasound-driven refinement of analogous hydroxypropyl-β-cyclodextrin further improves redispersibility and photothermal stability, thereby enhancing intestinal bioaccessibility without compromising antioxidant activity [70]. Lipid-based systems constitute a second lineage, bilosomes fortified with bile salts maintain over 70% EGCG integrity during simulated gastrointestinal digestion and increase oral bioavailability 1.98 times by exploiting endogenous bile acid transport pathways [71], whereas gelatinized-core liposomes embed EGCG within a thermoreversible gelatin network, markedly increasing its entrapment efficiency and sustaining intestinal release, thereby enhancing transepithelial permeability across Caco-2 monolayers and augmenting cellular antioxidant activity [72]. A third category embraces hybrid and stimuli-responsive constructs that respond to pH or redox gradients intrinsic to pathological tissues; alginate hydrogels coencapsulating EGCG-Mn nanoclusters and probiotics release their payload specifically within the inflamed colonic lumen, thereby reinforcing barrier integrity and modulating microbial metabolite profiles [73], whereas EGCG-cystamine nanoparticles exploit the elevated glutathione levels characteristic of atherosclerotic plaques, which selectively cleave the disulfide bonds within the particles and thus trigger site-specific disassembly and liberation of EGCG at the lesion site [74]. Importantly, these carriers not only augment systemic exposure but also preserve, and in some cases amplify, the EGCG-induced shifts in microbial composition and short-chain fatty acid (SCFA) production that are central to its remote organ effects. Collectively, the integration of cyclodextrin–protein scaffolds, lipidic shuttles and smart hybrid platforms is transforming EGCG from a labile dietary constituent into a precisely deliverable bioactive capable of orchestrating systemic homeostasis through the gut–organ axis.

## Role of EGCG in the Gut

The intestine, the largest immune organ, is crucial for digestion, absorption, and health regulation. Gut microbiota, bridging external stimuli like diet and the host, modulates physiological and biochemical functions linked to the gut barrier and disease. There is growing evidence that EGCG and its metabolites alter the abundance of gut microbiota, mainly by promoting the proliferation of beneficial bacteria such as Akkermansia, *Akkermansia* and *Bifidobacterium* and inhibiting the growth of harmful ones like *Desulfovibrio* [75,76], enhancing gut barrier integrity via the production of SCFAs and activation of the AhR pathway [77,78], and reducing gut inflammation by suppressing the TLR4/NF-κB signaling cascade [78,79].

### Regulation of defects in gut barrier function by EGCG

A good intestinal barrier, which is crucial for maintaining intestinal homeostasis and a prerequisite for the normal development of gut microbiota [80], and the gut microbiota and their metabolites located near the gut epithelium can affect the intestinal mechanical and chemical barriers. The gut microbiota can influence tight junction proteins (TJPs) expression, induce apoptosis or proliferation of IECs to regulate the intestinal mechanical barrier [81], and affect mucus layer formation to regulate the intestinal chemical barrier [80]. For example, Pontarollo et al. [82] demonstrated that the gut microbiota, via activation of epithelial Toll-like receptor 2 (TLR2), suppresses Hedgehog (Hh) signaling by inducing lysosomal degradation of IEC-expressed neuropilin-1, which leads to downregulation of TJPs and a weakened gut barrier. The validity of this mechanism is contingent on the presence of microbial ligands capable of activating the TLR2/TLR6 pathway.

Numerous animal experiments have demonstrated that EGCG enhances intestinal barrier function by upregulating TJPs like Zonula occludens-1 (ZO-1) and Occludin and increasing intestinal stem cells and crypt cells [39,[83], [84], [85]], reducing intestinal mucosa damage from external factors [19]. Mechanistically, EGCG reinforces the intestinal barrier by simultaneously reshaping the microbiota and reactivating epithelial-protective signaling. It increases the abundance of probiotics *Faecalibaculum* and *Akkermansia*, which are associated with enhanced mucus layer thickness and barrier integrity, and reduces the abundance of pathogenic microorganism such as *Tuzzerella*, thereby mitigating microbial imbalance and inflammation [77]. The reduction in pathobionts like *Tuzzerella* helps suppress the activation of the TLR4/NF-κB signaling pathway, a major driver of proinflammatory cytokine production (TNF-α, IL-6). This improved microbial environment promotes the restoration of L-glutamine levels. L-glutamine serves as a primary energy source for IECs and supports barrier function by stimulating the expression of TJPs (ZO-1, Claudins) and activating the AhR-IL-22 pathway, a key axis for maintaining epithelial homeostasis and repair [77]. EGCG selectively enriches *Lactobacillus* and *Bifidobacterium*—2 genera harboring butyryl-CoA and acetate-CoA transferase genes—thereby enhancing microbial fermentation of dietary fibers into the SCFAs acetate, propionate and butyrate, although simultaneously suppressing *Desulfovibrio* and *Bacteroides* that lack these biosynthetic enzymes, resulting in reduced LPS release and restored intestinal barrier integrity [78]. EGCG also activates the Nrf2 pathway, enhancing TJPs expression like ZO-1 and occludin, further reinforcing the intestinal barrier [78]. Hypoxia-inducible factor-1α (HIF-1α) is key for protecting the intestinal barrier by coordinating various target genes for intestinal epithelial barrier integrity, and EGCG intervention can restore dysregulated levels of HIF-1α expression [84]. Dey et al. [86] found EGCG maintained HIF-1α and TJP mRNA expression in the ileum and colon of mice with nonalcoholic steatohepatitis. Moreover, Akkermansia can regulate intestinal barrier function through the outer membrane protein Amuc_1100, a key mediator that strengthens tight junctions and reduces gut permeability by interacting with host TLR2, and bacterial extracellular vesicles, which deliver bioactive molecules to host cells to modulate immune responses and promote epithelial integrity [87]. Cheng et al. [84] demonstrated EGCG increases the growth of beneficial bacteria, such as *A. muciniphila* enhancing the synthesis of mucins (MUC2), to form a protective mucus layer over the intestinal epithelium, maintaining gut barrier function and safeguarding it from pathogens. Additionally, EGCG increases the abundance of beneficial bacteria like *Dubosiella* and *Akkermansia*, boosting the mRNA levels of ileal TJPs, including ZO-1, Occludin, and junctional adhesion molecule-A, promoting antimicrobial peptide expression, and strengthening the intestinal barrier function [87].

### Regulation of gut inflammatory response by EGCG

Inflammatory response can significantly alters gut physiology [88] which causes changes in proinflammatory gene protein expression, TJP, and free fatty acid receptors, and ultimately impairing gut barrier function. Conversely, stable gut barrier function can reduce the frequency of inflammation. Cani et al. [89] showed gut microbiota changes such as an increased abundance of *Bifidobacterium*, *Lactobacillus* and the *C. coccoides–Eubacterium*, can increase intestinal proglucagon mRNA and endogenous glucagon-like peptide-2 (GLP-2) secretion, improving gut barrier function, and alleviates inflammation.

EGCG, with its anti-inflammatory and antioxidant properties, is widely used in inflammatory bowel disease dietary studies. For example, Yeoh et al. [90] found EGCG can effectively inhibit myeloperoxidase (MPO) activity. Che et al. [83] discovered EGCG reduces inflammation by suppressing Th1 cell polarization and downregulating proinflammatory cytokines like IFN-γ in mouse colitis models. Wu et al. [91] also found EGCG reduces macrophage and neutrophil infiltration and downregulate the expression of genes related to inflammatory response and immune modulation.

Beyond direct anti-inflammatory effects, EGCG modulates gut microbiota to inhibit gut inflammation. It increases beneficial bacteria like *Lactobacillaceae* and *Lachnospiraceae* improving the gut inflammatory network by regulating regulatory T (Treg) cells, among other things [92], whereas decreases the harmful bacteria like *Enterococcaceae* and *Enterobacteriaceae*. In Chen et al.’s [84] study on *Salmonella* infection in mice, EGCG interacts with beneficial bacteria like *A. muciniphila* to inhibit inflammatory pathways such as the TLR4/MyD88/NF-κB. It can also reduce the abundance of harmful bacteria such as *Desulfovibrio*, linked to increased LPS production, thereby controlling the inflammatory response induced by high-fat diets (HFDs) and Salmonella infection [84]. In addition, EGCG increases the abundance of *Dubotella* which is negatively correlated with various inflammatory factors, thus inhibiting the expression of IL-1β and other inflammatory factors [87].

### Regulation of SCFAs by EGCG

**FIGURE 2:**
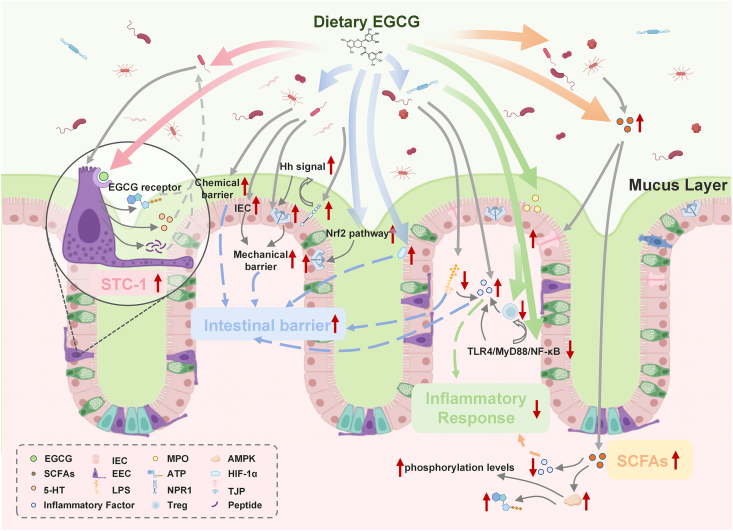
EGCG in modulating intestinal homeostasis: (A) gut barrier protection: EGCG reshapes gut microbiota and activates Nrf2 pathway to strengthen tight junctions and mucus layer. (B) Anti-inflammatory effects: EGCG suppresses TLR4/NF-κB signaling and reduces inflammatory factor via microbiota modulation. (C) SCFA metabolism: EGCG enriches SCFA-producing bacteria, elevating butyrate/acetate to activate AMPK/AhR-IL-22 and reinforce barriers. (D) Microbiota-synergistic interactions: EGCG is metabolized by gut microbes into bioactive compounds that regulate detoxification and hormone signaling via EC-STC-1 feedback loops. 5-HT, 5-hydroxytryptamine; AMPK, AMP-activated protein kinase; EEC, enteroendocrine cell; EGCG, epigallocatechin gallate; Hh, Hedgehog signaling pathway; IEC, intestinal epithelial cell; MPO, myeloperoxidase; MyD88, myeloid differentiation primary response 88; NF-κB, nuclear factor kappa B; NPR1, neuropilin-1; Nrf2, nuclear factor erythroid 2-related factor 2; SCFAs, short-chain fatty acids; STC-1, secretin tumor cell line-1; TLR4, Toll-like receptor 4; TJP, tight junction protein.

SCFAs are the primary metabolites of most gut bacteria and serve as a crucial link between the gut microbiota and the host [93]. In human gut, SCFAs mainly consist of acetate, propionate, and butyrate, where acetate is a net fermentation product, butyrate is often derived by microorganisms based on acetate or lactate via the glycolytic pathway [94]. As shown in Figure 2, SCFAs have the functions of improving lipid metabolism, anti-inflammation, and protecting the gut barrier [95], and can modulate ≤20% of the body's gene expression [96]. Donohoe et al. [97] found that SCFAs were able to activate the AMPK process and improve phosphorylation levels and ATP levels. It was found that SCFAs were able to upregulate anti-inflammatory and downregulate proinflammatory cytokines through different mechanisms, respectively, leading to global anti-inflammatory effects [98]. Additionally, SCFAs modulate occludin, bandins, and claudins to decrease intestinal permeability, thereby increasing the degree of IEC tight junctions [[99], [100], [101]]. Numerous experiments have demonstrated the ability of EGCG to enhance the levels of SCFAs in the gut shown in Table 1 [10,19,39,78,79,84,85,87,88,92,93,[103], [104], [105], [106]]. Wu et al. [91] reported that oral administration of EGCG increased the levels of SCFAs in the gut of mice infected with *C. difficile* by determining the concentration of cecum contents. Wu et al. [92] also found that EGCG enriched SCFA-producing bacteria such as *Akkermansia*, which subsequently increased SCFA production and ameliorated colitis. In a very recent study, Zuo et al. [78] demonstrated that EGCG intervention significantly increased the abundance of 6 SCFA-producing bacterial groups in HFD-fed rats, including *Lactobacillus, Ruminococcus_1, Clostridium,* and *Akkermansia*. Although Che et al. [83] found that EGCG can alleviate dextran sulfate sodium-induced colitis in mice by increasing the abundance of beneficial bacteria, such as *Bacteroides*, the main bacterial group that produces propionate in the gut, and *Akkermansia*, which produces acetate and propionate. In a study on HFD mouse model, Ma et al. [87] found that EGCG increases the abundance of *Dubosiella* and *Akkermansia*, thereby significantly elevating the levels of SCFAs, contributing to the regulation of metabolic diseases.

**TABLE 1:** Protective effect of EGCG in the gut.

	Model	Dosages regimen	Specific impact	Microflora affected	Reference
In vitro	In vitro human fecal	Human fecal suspension was mixed with 0.1 mmol/L EGCG for 48 h	Changes in concentrations of EGCG and its metabolites were strongly correlated with changes in the abundance of specific gut microbiota.	Abundance of *Bacteroides*, *Christensenellaceae*, and *Bifidobacterium* ↑ Abundance of *Fusobacteriumvarium*, *Bilophila*, and *Enterobacteriaceae* ↓	[10]
In vitro	Caco-2 and in vitro fermentation	1% (w/v) GTP/OTP/BTP polyphenols; 50 μg/mL for Caco-2	Antioxidant, GTP > OTP > BTP; low papp and efflux ratios	Abundance of *Bifidobacterium*, *Lactobacillus*, and *Enterococcus* ↑ Abundance of *Bacteroides–Prevotella* and *Clostridium histolyticum* ↓	[102]
Animal Study	C57BL/6 female mice	*Clostridium difficile* infection mice were fed with 25/50 mg/kg body weight EGCG for 14 d	Structural damage to the intestine ↓ The mRNA expression levels of ZO-1, Occludin and MUC2 ↑ The serum levels of LBP, IL-1α, IL-1β, IL-6, TNF-α, MIP-1α, and MCP-1 ↓ amino acids such as L-aspartic acid ↓ The transcriptional downregulation of intestinal genes induced by *C. difficile*, including genes associated with lipid binding (Apol9a and Apol9b) and immune regulation (Ccl22, Alox15, Cxcl9, and C1qtnf3) ↓	The levels of *Lactobacillaceae* and *Lachnospiraceae* ↑ The levels of endotoxin-producing bacteria *Enterococcaceae* and *Enterobacteriaceae* ↓ SCFAs ↑	[91]
Animal study	C57BL/6J male mice	Total body irradiation (TBI) mice were fed with 25 mg/kg body weight EGCG before (once a day for 5 d) and 30 min after TBI	Intestinal mucosal injury ↓ preserve ISCs Intestinal epithelial cell regeneration ↑ The number of Lgr5+ intestinal stem cells (ISCs) and Ki67+ crypt cells↑	Abundance of *Firmicutes* ↑ *Firmicutes*/*Bacteroidetes* ratio ↑ Abundance of *Bacteroidales*, *Blautia*, *Turicibacter*, *Lactobacillus* ↑ Abundance of *Lactobacillus gasseri*, *L. murinus*, and *L. reuteri* ↑	[39]
Animal study	C57BL/6J male mice	TBI mice were intraperitoneally injected with 25 mg/kg body weight EGCG before (once a day for 5 d) and 30min after TBI	Mice’s survival time ↑ mice’s body weight ↑ The Ki67-positive and Lgr5-positive cells per crypt ↑ Proliferation and survival of intestinal stem cells ↑ The number of TUNEL-positive cells, γH2AX foci and 8-OHdG-positive cells ↓	—	[19]
Animal study	C57BL/6 female mice	DSS-induced colitis mice were fed with EGCG or rectally administrated with 50 mg/kg body weight EGCG for 3 d	Disease activity index ↓ The level of MPO, IL-1β, IL-6, IL-8 and TNF-α ↓ Total antioxidant capacity, total superoxide dismutases, CAT ↑ MDA ↓	Abundance of *Akkermansia*, *UBA1819*, *Lacknoclostridium*, *Colidextribacter* ↑	[92]
Animal study	Male C57BL/6 mice	Polystyrene microplastics mice fed with 50 mg/kg body weight EGCG for 35 d	Microbial diversity decreased after EGCG treatment. EGCG optimized the microbial composition mRNA expression levels of claudin5, ZO-1 and occludin ↑ The level of LPS, TNF-α,IL-6 and IL-1β ↓ EGCG remodeled serum metabolism, especially regulating purine metabolism. The TLR4/MyD88/NF-κB signaling cascade ↓	Abundance of *unclassified_f_Ruminococcaceae*, *Butyricicoccus*, and pathogenic microbiota *Tuzzerella* ↓ Abundance of probiotics *Faecalibaculum* and *Akkermansia* ↑	[77]
Animal study	Male SD rat	High-fat diet fed rat daily administration of 100 mg/kg and 200 mg/kg body weight EGCG by intragastric gavage for 8 wk	Inhibits intestinal barrier dysfunction and inflammation restores gut microbiota diversity and composition. Beneficial microbes, particularly SCFAs producers like *Lactobacillus*↑ Harmful Gram-negative bacteria, such as *Desulfovibrio*↓ Increases SCFAs levels and decreases LPS levels. The TLR4/NF-κB inflammatory pathway ↓ The Nrf2 pathway↑	Abundance of *Ruminococcaceae_UCG-014*, *Ruminococcus_1*, *Lactobacillus*, *Bifidobacterium*, and *Akkermansia* ↑ Abundance of *Bacteroides*, *Desulfovibrio*, *Lachnoclostridium*, *Lachnospiraceae_ND3007_groups*, *Parasutterella*, and *Phascolarctobacterium* ↓	[78]
Animal study	C57BL/6J male mice	DSS-induced colitis mice were gavaged with 80 mg/kg body weight EGCG for 21/24 d	The number of CD4 T cells↓ macrophage populations ↑ The expression of marker genes for Th1, Th17, and Treg cells ↓ beneficial probiotic strains, particularly SCFA-producing bacteria like *Lachnoclostridium*, *Akkermansia*, and *Bacteroides* ↑ The concentration of SCFAs in the gut ↑ The GPR43 receptor on intestinal epithelial cells ↑ IFN-γ secretion from Th1 cells↓ Th1 cells polarization and self-amplification↓ GPR43 expression in colonic tissues ↑	Abundance of *Lactobacillus*, *Akkermansia*, *Bacteroides*, *Ruminococcus*, and *Enterococcus* ↑ Abundance of *Helicobacter*, *Escherichia-Shigella*, and *Oscillibacter* ↓	[83]
	C57BL/6J female mice	*Salmonella* infection high-fat diet mice were gavaged with 300 mg/kg body weight EGCG for 4 wk	Intestinal barrier function↑ The synthesis of mucins (MUC2) ↑ The expression of tight junction proteins, such as ZO-1 and occluding ↑ The levels of proinflammatory cytokines, particularly TNF-α and IL-1β ↓ The growth of beneficial bacteria, such as *Akkermansia muciniphila* ↑ the proliferation of harmful bacteria like *Desulfovibrio* ↓	Abundance of *Akkermansia muciniphila*, *Candidatus Saccharimonas*, and *Firmicutes* ↑ abundance of *Desulfovibrio*, *Proteobacteria*, *Desulfovibrionaceae*, and *Enterobacteriaceae* ↓	[84]
	C57BL/6 male mice	High-fat diet mice with intragastric administration of 50 mg/kg EGCG for 12 wk	Body weight ↓ Dyslipidemia ↓ Glucose tolerance in diet-induced obese mice ↑ Abnormalities in liver function ↓ Stabilized hypoxia-inducible factor 1α (HIF1α) mRNA levels of ileal TJ proteins including ZO-1, Occludin, and JAM-A ↑ The expressions of antimicrobial peptides ↑ SCFAs contents ↑ The abundance of *Dubosiella* and *Akkermansia* ↑	The abundance of *Dubosiella* and *Akkermansia* ↑	[87]
Animal study	C57BL/6J male mice	Mice were provided a diet formulated to high-fat containing EGCG (0.3%) for 8 wk	Maintaine mRNA expression of ileal and colonic HIF-1α and TJPs fecal calprotectin ↓ the F,B ratio ↓	Abundance of *Firmicutes* ↑ Abundance of *Bacteroidetes*↓	[86]
Human study	50–80 y with resected colorectal adenomas (*n* = 632 mITT, *n* = 545 PP)	MIRACLE trial, RCT, 3 y intervention (GTE, 150 mg EGCG twice a day vs. placebo)	No sig. difference in adenoma recurrence (GTE 51.1% vs. placebo 55.7%, mITT; *P =* 0.161); benefit in males (GTE 52.9% vs. placebo 60.4%, mITT; *P* = 0.048), no benefit in females	—	[103]
Human study	Meta-analysis of human RCTs (no experimental model)	EGCG 300 mg/d (main dose) for 1–3 y (varied by 5 included RCTs)	Significantly reduced colorectal cancer/adenoma recurrence (pooled RR = 0.85, 95% CI: 0.76, 0.95, *P* < 0.05); more effective in Asians and males	—	[104]
Human study	Human clinical model (RCT)	EGCG 150 mg twice daily (total 300 mg/d) for 3 y; 1-mo run-in period	Trend of reduced colorectal adenoma recurrence (EGCG 51.1% vs. placebo 55.7%, modITT; adj *P =* 0.077); well-tolerated (no major AE differences)	—	[105]

Abbreviations: 8-OHdG, 8-hydroxy-2′-deoxyguanosine (oxidative-DNA adduct); γH2AX, phosphorylated histone 2AX (DNA-damage marker); AE, adverse event; BTP, Black tea polyphenols; CAT, catalase; CYP450, Cytochrome P450; DSS, dextran sulfate sodium; EGCG, epigallocatechin gallate; GTP, green tea polyphenols; GTE, green tea extract; HIF-1α, hypoxia-inducible factor 1-alpha; ISCs, intestinal stem cells; JAM-A, Junctional adhesion molecule-A; LBP, LPS-binding protein; MCP-1, Monocyte chemoattractant protein-1; MDA, Malondialdehyde; MIP-1α, Macrophage inflammatory protein-1 alpha; MIRACLE, Minimizing the Risk of Metachronous Adenomas of the Colorectum with Green Tea Extract; modITT, modified intention-to-treat; MPO, Myeloperoxidase; MUC2, mucin 2; mITT, modified intention-to-treat; NF-κB, nuclear factor kappa B; Nrf2, nuclear factor erythroid 2–related factor 2; OTP, Oolong tea polyphenols; RCT, randomized controlled trial; SCFAs, short-chain fatty acids; TBI, total body irradiation; TJPs, tight junction proteins; TLR4, Toll-like receptor 4; TUNEL, terminal deoxynucleotidyl transferase dUTP nick-end labelling; ZO-1, Zonula occludens-1.

### Beneficial synergistic interactions between EGCG and gut microbiota

Gut microbes are closely linked to immune and metabolic disorders in humans. They maintain human health by interfering with gut dynamics, regulating energy metabolism and promoting human immunity [101,106]. On the basis of natural properties, dozens of phyla of bacteria have been identified in human intestinal flora, almost 98% of which are attributed to the phyla of *Bacteroides*, *Actinobacteriota*, *Firmicutes*, and *Proteobacteria*. At the genus level, *Bifidobacterium* and *Lactobacillus* are the dominant species which make an indispensable contribution to intestinal health [107]. They are distributed in the large intestine, where they encounter cells, exchange substances and transmit energy and information. Under normal conditions, they maintain a balanced state regarding species and numbers, build the intestinal biological barrier and assist the intestinal immune system [108].

The interaction between EGCG and gut microbiota has been extensively studied and verified through animal models [109,110]. Many experiments have shown that the efficiency of metabolic utilization of dietary polyphenols depends on the composition of gut microbiota [[111], [112], [113]]. When the balance of intestinal microorganisms is disrupted by antibiotics, the usefulness of EGCG and its metabolites during the metabolism of dietary EGCG is also reduced [114]. Meanwhile, both in vivo and in vitro experiments have demonstrated that EGCG significantly alters the diversity and composition of the gut microbiota and enriches certain gut microbiota, such as *Flavonifractor*, which are flavonoid degrading gut bacteria to regulate various gut microbiota metabolites. The affected gut microbiota further participates in the transformation of EGCG and produces new bioactive components [52,115].

Additionally, gut microbiota transforms EGCG into bioactive metabolites that mediate its health effects. Pyrogallol (PG), derived from microbial degradation of GA, activates the Nrf2 pathway with significantly higher potency than EGCG—its 5% benchmark dose (BMD_5_) is 0.35 μM compared with 2.45 μM for EGCG [116,117]. Another key metabolite, 4′-amino-EGCG (4′-NH_2_-EGCG), is formed via microbial oxidation of EGCG to a quinone intermediate, followed by conjugation with ammonia [118]. 4′-NH_2_-EGCG neutralizes toxic endogenous compounds such as reactive carbonyl species such as methylglyoxal and ammonia, offering dual detoxification benefits while retaining anti-inflammatory and anticancer activities [118]. EGCG-4″-sulfate, the major phase II metabolite formed by SULT (SULT1A3 in the gut), prolongs EGCG’s circulation and serves as a reservoir for active EGCG in tissues [119]. However, the metabolic outcomes of EGCG are dose-dependent—high-dose EGCG (>750 mg/kg) leads to accumulation of hepatotoxic quinone metabolites, whereas chronic low-dose intake promotes detoxification pathways [118]. Human studies confirm that daily consumption of 4 cups of green tea increases urinary excretion of EGCG-amino and EGCG-reactive carbonyl conjugates, highlighting the functional relevance of this microbiota–metabolite–detoxification axis [118].

Moreover, enteroendocrine cell (EEC) plays a significant role in the metabolism influenced by the gut microbiota. As shown in Figure 2, they detect the presence of nutrients, microbiota, and their metabolites, then send relevant signals such as 5-hydroxytryptamine (5-HT), ATP, and peptides, and peptides back to the gut microbiota. Greiner et al. [120] found that the gut microbiota can specifically regulate the number of EEC population or choose to release SCFAs directly. Kurogi et al. [12] discovered that EEC secretin tumor cell-1 (STC-1) has receptors on its surface that recognize EGCG, which activates STC-1 and drives them to form actin stress fibers. These cytoskeletal structures enhance cell integrity and are often associated with cellular responses to stimuli, potentially contributing to hormone secretion [121]. Because STC-1 cells are associated with hormones related to metabolism, feeding, and satiety [122], the stimulation process of EGCG on STC-1 cells affects the signaling feedback of EEC to the gut microbiota, further highlighting the close and complex interaction between EGCG and gut microbiota.

## Interconnected EGCG–Gut Axes and Systemic Health

### EGCG–gut–liver axis

The liver is involved in numerous physiological processes within the body and is vital to human life. It is responsible for the metabolic regulation of nutrients, in vivo homeostasis of lipids and cholesterol, and catabolism of xenobiotic compounds [123]. The gut and the liver have a close connection, with the bidirectional interference of nutrients and metabolites between them is realized through the portal system [124].

There is growing evidence that EGCG and its metabolites can improve liver disorders by synergizing with the gut microbiota, in addition to directly intervening in hepatocytes, lipid droplet accumulation and glycogen synthesis processes [77,[125], [126], [127]].

#### Regulation of hepatic glucose metabolism by EGCG–gut microbiota

**FIGURE 3:**
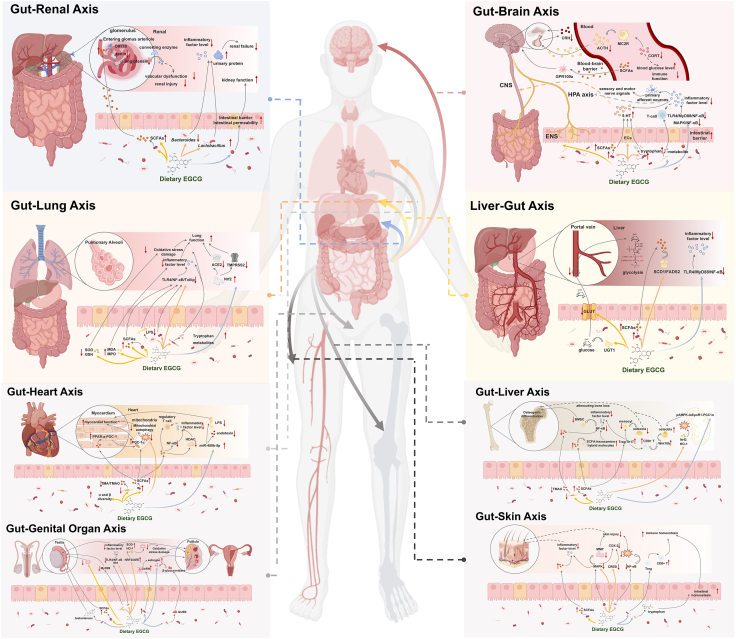
Schematic of EGCG–gut microbiota synergistic regulation of multiorgan axes: (A) EGCG–gut–liver axis: EGCG reshapes gut microbiota, reduces LPS translocation via the portal system, and activates hepatic PPAR-α/SCFAs to improve glucose and fatty acid metabolism whereas suppressing TLR4/NF-κB-driven inflammation. (B) EGCG–gut–brain axis: EGCG reshapes gut microbiota, activates SCFAs/GPR109a signaling, and inhibits TLR4/NF-κB to reduce neuroinflammation, although modulating HPA axis activity and 5-HT homeostasis to alleviate stress and depressive behaviors. (C) EGCG–gut–renal axis: EGCG modulates gut microbiota, elevates SCFAs to inhibit RAS/ACE activity and Nrf2/Keap1 antioxidant pathways, although promoting urate excretion via OAT gene regulation and suppressing renal inflammation to ameliorate nephropathy. (D) EGCG–gut–lung axis: EGCG reshapes gut microbiota (↑ *Bifidobacterium*, ↓ *E. coli*) to enhance SCFAs production, inhibit NF-κB/NLRP3 inflammation, and strengthen gut barrier, indirectly reducing lung inflammation and alleviating COPD/asthma via the gut–lung axis. (E) EGCG–gut–heart axis: EGCG reshapes gut microbiota, inhibits NF-κB/LPS-driven inflammation, and reduces oxidative stress to ameliorate heart failure and cardiac dysfunction via the gut–heart axis. (F) EGCG–gut–skin axis: EGCG promotes beneficial gut microbiota growth, enhances gut barrier, and suppresses COX-2/p38 MAPK inflammation to alleviate UV-induced skin damage and acne via the gut–skin axis. (G) EGCG–gut–bone axis: EGCG modulates gut microbiota, reduces oxidative stress, and regulates hormone metabolism (e.g., E2, Cyp19) to protect ovarian/testicular function and alleviate infertility. and (H) EGCG–gut–reproductive axis: EGCG enriches beneficial gut microbiota, repairs gut barrier (↑ ZO-1/Claudin-1), and inhibits osteoclast activity to increase trabecular bone density and combat osteoporosis via the gut–bone axis. 5-HT, 5-hydroxytryptamine; ACE2, angiotensin-converting enzyme 2; ACTH, adrenocorticotropic hormone; AKT, protein kinase B; AMPK, AMP-activated protein kinase; BBB, blood-brain barrier; BMSC, bone marrow stromal cell; CD8+ T, CD8-positive T lymphocyte; CNS, central nervous system; COPD, chronic obstructive pulmonary disease; CORT, corticosterone; COX-2, cyclooxygenase-2; CREB, cAMP response element-binding protein; CRH, corticotropin-releasing hormone; ECs, enterochromaffin cell; EGCG, epigallocatechin gallate; ENS, enteric nervous system; FADS2, fatty acid desaturase 2; GLUT, glucose transporter; GnRH, gonadotropin-releasing hormone; GPR109a, G protein-coupled receptor 109a; HDAC, histone deacetylase; HO-1, heme oxygenase-1; HPA,hypothalamic-pituitary-adrenal; MAPK, mitogen-activated protein kinase; MC2R, melanocortin 2 receptor; MDA, malondialdehyde; miR-450b-5p, microRNA-450b-5p; MMP, matrix metalloproteinase; MPO, myeloperoxidase; MyD88, myeloid differentiation primary response 88; NF-κB, nuclear factor kappa B; Nrf2, nuclear factor erythroid 2-related factor 2; Olfr78, olfactory receptor 78; pAMPK, phosphorylated AMP-activated protein kinase; PGC-1α, peroxisome proliferator-activated receptor gamma coactivator 1-alpha; PPARα, peroxisome proliferator-activated receptor alpha; SCD1, stearoyl-CoA desaturase 1; SCFAs, short-chain fatty acids; SOD, superoxide dismutase; T cell, T lymphocyte; Th17, T helper 17 cell; TLR4, Toll-like receptor 4; TMA, trimethylamine; TMAO, trimethylamine N-oxide; TMPRSS2, transmembrane protease, serine 2; Tollip, Toll-interacting protein; Treg, regulatory T cell; UGT1, UDP-glucuronosyltransferase family 1; Wnt10b, Wnt family member 10B; ZO-1, Zonula occludens-1.

The liver can regulate blood sugar levels through glycogen synthesis, catabolism, storage and release. It takes up carbohydrates from the intestines via the portal vein. In hepatocytes, glucose is phosphorylated to enter the cells fully for metabolism or glycogen synthesis [128]. However, excessive glucose intake increases the release of gut inflammatory factors and the number of adhesion molecules, which will also act on the liver through the portal vein, inducing gut and liver dysfunction. EGCG has been proven to effectively inhibit the activity of α -glucosidase and reduces the release of α-glucose molecules, thereby suppressing the increase of blood glucose levels in the body [129]. As shown in Figure 3, oral EGCG exert regulatory effects through intestinal metabolism and hepatic circulation. Sun et al. [130] compared the regulatory effects of green tea extract, catechins and EGCG on hepatic metabolism in mice fed a HFD and found the effect of green tea extracts on the metabolic and phenolic profiles of the gut microbiota was found to be more dependent on EGCG, which occupies an important position in the modulation of gut microbiota diversity by tea-based diets. EGCG tends to regulate the process of glucose metabolism by mediating the glycolysis/gluconeogenesis metabolic pathway in the liver [131]. Wang et al. [132] found that EGCG-rich Pu-erh and Dian Hong tea polyphenols (with higher EGCG) enrich gut *Lactobacillus* to reduce the translocation of harmful microbial metabolites to the liver via the gut–liver axis, while also enhancing hepatic antioxidant capacity [elevated superoxide dismutase (SOD) activity and total antioxidant capacity] and regulating lipid metabolism-related genes; these effects collectively support glucose homeostasis and protect the liver. Ma et al. found that EGCG can restore the ileum barrier damaged by an HFD by regulating the abundance of Dubosiella and Akkermansia and enhancing the expression of TJPs and antimicrobial peptides. This improvement in barrier function helps to reduce metabolic endotoxemia (such as LPS translocation), thereby alleviating systemic and adipose tissue inflammation, which are key contributors to insulin resistance, ultimately leading to improved glucose metabolism disorders [87]. Furthermore, Zeng et al. [115] found that EGCG can regulate glycolipid metabolism and alleviate liver tissue damage caused by obesity by regulating the relative abundance of *A. muciniphila* in mice and the related LPS/insulin resistance. This leads to a reduction in circulating LPS levels, thereby ameliorating LPS-induced systemic and hepatic inflammation, which in turn improves insulin signaling such as via the PI3K/AKT pathway and reduces insulin resistance, ultimately promoting better glucose uptake and lipid homeostasis [115].

#### Regulation of fatty acids homeostasis in the liver by EGCG–gut microbiota

Human lipids are composed of fats and lipids, where fats are composed of fatty acids and glycerol. The presence of large amounts of free fatty acids in the host can induce lipotoxicity and cause organ damage or dysfunction [133]. In vitro tests in rodents have demonstrated that SFAs induce lipotoxicity in some cells, whereas unsaturated fatty acids are not toxic and may even be protective [134].

Numerous experimental evidences indicate that EGCG dietary intervention can increase unsaturated fatty acids content and decrease SFAs content [78,79,87,132]. EGCG regulate the expression of the fatty acid desaturase SCD1/FADS2 in the liver by directly inhibiting or by interfering with SCFAs, both of which are key regulatory enzymes for hepatic lipogenesis and play important roles in the synthesis of unsaturated fatty acids [135]. Additionally, EGCG intervention also increased the expression of the fatty acid catabolic enzyme hormone-sensitive lipase [87]. Zhang et al. [79] explored the mechanisms by which EGCG alleviates bisphenol A (BPA)-induced metabolic disorders, showing that EGCG reduces the mRNA expression of genes related to fatty acid (Elov16) and cholesterol (CYP4A14) synthesis, whereas increasing the expression of fatty acid oxidation genes (Lss). These changes lead to a reduction in liver weight and total cholesterol levels, as well as improved cholesterol metabolism (Cyp7a1) and normalized BPA-induced gut microbiota dysbiosis.

In addition, EGCG promotes the growth abundance of specific bacteria such as *Dubosiella* and *Akkermansia*, which promotes the production of unsaturated fatty acids and decreases the production of SFAs [87]. As shown in Table 2 [24,77,79,80,87,116,127,131,132,[138], [139], [140], [141], [142],^237^], EGCG alleviate liver injury by promotes the growth of beneficial microorganisms, such as *Lactobacillus*, *Parabacteroidesis*, and *Akkermansia*, which are positively correlated with SCFAs, and inhibits the growth of Gram-negative bacteria like *Desulfovibrio* (X. Liu et al., 2021) [78,132,139]. These bacteria are vital for the production of SCFAs, particularly butyrate. Butyrate activates peroxisome proliferator-activated receptor-α, a nuclear receptor that regulates fatty acid oxidation and energy metabolism, thereby contributing to improved gut barrier function and reduced inflammation [132]. EGCG has also been shown to regulate the metabolism of glycerophospholipid, glycerolipid and sphingolipid to regulate lipid metabolism disorders and improving hepatic steatosis [139].

**TABLE 2:** Protective effect of EGCG–gut microbiota in the liver.

	Model	Dosages regimen	Specific impact	Microflora affected	Reference
Animal study	C57BL/6J male mice	High-fat diet fed mice supplemented with 0.3% EGCG for 8 wk	Compared with catechins, aspects that be fully prevented only by green tea extract and EGCG: obesity and insulin resistance ameliorate NASH pathology endotoxin–TLR4–NF-κB inflammation ↓ mRNA expression levels of claudin-1, ZO-1 and occludin ↑ expression of TJPs and HIF-1α	Abundance of *Anaeroplasmatales, Bacteroidetes*, *Verrucomicrobia*, *Akkermansia* and *Coriobacteriales*↑ Abundance of *Clostridiales*, *Mollicutes RF39*, *Ruminiclostridium*, *Clostridium cluster 1 Acetatifactor*, *Lachnoclostridium* and *LachnospiraceaeUCG-006* ↓	[76]
Animal study	C57BL/6J Male mice	Methionine–choline-deficient diet fed mice gavaged with 50 mg/kg of EGCG for the 2 wk	Reduced body weight loss and liver weight ratioregulated serum blood glucosealleviated hepatic injury, lipid accumulation, fibrosis progression, iron-ion loading, and plasma parametersregulate microbiota imbalance	Abundance of *norank_f__Bacteroidales_S24_7_group*、*Alloprevotella and Bacteroides* ↑ Abundance of *Oxalobacter*、*Oscillibacter*、*Coprococcus_1* and *Desulfovibrio* ↓	[126]
Animal study	C57BL/6J Male mice	High-fat diet fed mice supplemented with 100 mg/kg body weight EGCG for 14 wk	Under the influence of EGCG SFAs ↓ unsaturated fatty acids ↑ the liver protein expression of SCD1 and FADS2 ↓ There was a strong positive correlation between abundance of *Bacteroides*, *Candidatus_Saccharimonas*, *Akkermansia* and serum fatty acids profile. *Akkermansia* content was positively correlated with unsaturated fatty acids and negatively correlated with SFAs.	Abundance of *Verrucomicrobia* ↑ Abundance of *Firmicutes* and *Saccharibacteria* ↓	[136]
Animal study	Male Swiss albino mice	Mice were fed with 100 mg/kg body weight for 6/10/14/18 mo	EGCG consumption improves animal survival improves cellular autophagic response the expression of p53 and p21 in organs (intestine and liver) and tissues (adipose tissue) ↓ AMPK and AKT activation ↓ SIRT3 and SIRT5 expression ↑ the surface expression of early T cell activation molecule (CD69) in 18 mo old animals ↑ the levels of IL-1β/TNF-α in 18 mo old animals ↓ relative gene expression of TNF-α in 14 mo old animals ↓	Preserve microbial diversity Abundance of *Clostridium, Staphylococcus, Streptococcus* ↓	[137]
Animal study	C57BL/6J male mice	Polystyrene microplastics mice supplemented with 50 mg/kg body weight EGCG for 14 d	EGCG did not alter the microbial richness, but increased abundance of probiotics. EGCG repressed the TLR4/MyD88/NF-κB pathway ZO-1, Occludin and Claudin5 at the protein level ↑ The level of LPS, TNF-α,IL-6 and IL-1β ↓ The level of ALT, AST, ALP and LDH ↓ regulation of fibrosis-related genes, such as α-SMA, Col1a1, and Col3a1 ↑	Abundance of *Akkermansia*, *Mucispirillum*, and *Faecalibaculum* ↑ Abundance of *Butyricicoccus*, *Ruminococcus*, and pathogenic microbiota *Tuzzerella* ↓	[138]
Animal study	C57BL/6J male mice	Mice were provided a diet formulated to high-fat containing EGCG (0.3%) for 8 wk	Adiposity ↓ NASH pathology (steatosis and ballooning) ↓ Normalize liver pathology serum endotoxin and hepatic TLR4/NFκB inflammation↓ Maintain mRNA expression of ileal and colonic HIF-1α and TJPs Fecal calprotectin ↓ The F,B ratio ↓	Abundance of *Firmicutes* ↑ Abundance of *Bacteroidetes*↓	[86]
Animal study	Male SD rat	High-fat diet fed rat daily administration of 100 mg/kg and 200 mg/kg body weight EGCG by intragastric gavage for 8 wk	EGCG improves NAFLD phenotypes and metabolic disorders in rats on a high-fat diet (HFD) liver steatosis and inflammation↓ inhibits intestinal barrier dysfunction and inflammation restores gut microbiota diversity and composition. Beneficial microbes, particularly short-chain fatty acid (SCFA) producers like *Lactobacillus*↑ harmful Gram-negative bacteria, such as *Desulfovibrio*↓ increases SCFA levels and decreases LPS levels. The TLR4/NF-κB inflammatory pathway ↓ The Nrf2 pathway↑	Abundance of *Ruminococcaceae_UCG-014*, *Ruminococcus_1*, *Lactobacillus*, *Bifidobacterium*, and *Akkermansia* ↑ Abundance of *Bacteroides*, *Desulfovibrio*, *Lachnoclostridium*, *Lachnospiraceae_ND3007_groups*, *Parasutterella*, and *Phascolarctobacterium* ↓	[78]
Animal study	Female ICR mice	Bisphenol A treated mice were administered 20 mg/kg or 60 mg/kg body weight EGCG for 12 wk	Bisphenol-A (BPA)-induced lipid metabolism disorders↓ The normalization of gut microbial dysbiosis caused by BPA↑ Improved lipid metabolism by regulating amino acid and fat biosynthesis Reduced body weight, liver weight ratio, and triglyceride reduced cholesterol levels by altering gene expression related to fatty acid synthesis (Elov16), cholesterol synthesis (CYP4A14), fatty acid oxidation (Lss), and cholesterol metabolism (Cyp7a1) influence the biosynthesis of L-cysteine, glycerophosphorylcholine, and palmitoleic acid	Abundance of *Bacteroidetes* ↑ Abundance of *Firmicutes*, *Lactobacillus*, *Corynebacterium* and *Staphylococcus* ↓	[79]
Animal study	C57BL/6J male mice	High-fat diet fed mice supplemented with 0.3% EGCG for 8 wk	EGCG plays an important role in the gut with an anti-obesity potential modulated by metabolic regulation independent from its impact on microbial population. The abundance of N-acetylserotonin ↑	Abundance of *Akkermansia*, *Bacteroidetes, Betaproteobacteriales*, *Anaeroplasmatales*, *Parasutterella*, *Proteobacteria*, *Verrucomicrobia*, and *Verrucomicrobiales* ↑ Abundance of *Firmicutes* ↓	[130]
Animal study	C57BL/6J male mice	High-fat diet mice were provided diet of 80 mg/kg EGCG for 4 wk	Alleviate obesity Regulate glycolipid metabolism Reduce hepatic steatosis Lower serum lipopolysaccharide (LPS) levels The abundance of Akkermansia muciniphila↑	Abundance of *Akkermansia muciniphila*↑	[115]
Animal study	Leptin receptor-knockout male rat	EGCG at a dose of 100 mg/kg/d was administered through oral tube feeding for 24 wk	Regulate glycerophospholipid, glycerolipid and sphingolipid metabolism Activate the SIRT6/SREBP1/FAS pathway Reduce hepatic lipid accumulation Maintaining homeostasis of gut microbiota	Abundance of *Anaerobacterium*, *Clostridium_XVIII*, *Clostridium_sensu_stricto* and *Paraprevotella* ↑ Abundance of *Enterorhabdus* and *Streptococcus* ↓	[139]
Animal study	Sprague–Dawley male rat	Hyperlipidemia rats with intragastric administration of 200, 400, 800 mg/kg EGCG for 12 wk	Reduce the glycerophospholipids improve liver steatosis Reduce the level of aspartate aminotransferase Increase the abundance of beneficial bacteria	Abundance of *Parabacteroides*, *Bacteroides*, *Akkermansia*, *Butyricimonas*, *Desulfovibrio* and *Blautia*↑	[24]
Animal study	Wistar male rats	EGCG at a dose of 160 mg/kg/d was gavaged	Promote insulin secretion and increase serum insulin content lower blood glucose levels and improve insulin resistance protected systemic oxidative stress homeostasis to ease liver damage and kidney damage	Abundance of *Bacteroidaceae*, *Phocaeicola* and *Akkermansiamuciniphila*↑ Abundance of *Lachnospiraceae*, *Desulfovibrionaceae* and *Roseburia*↓	[131]
Human study	Randomized open-label cohort (pre-FRIEND); 18–40 y reproductive-aged women (with/without fibroids)	720 mg EGCG/d (with breakfast) for 30–35 d, EGCG alone; EGCG+clomiphene (100 mg/d, 5 d); EGCG+letrozole (2.5 mg/d, 5 d)	No liver injury (no Hy’s law cases); mild AEs (GI/insomnia); normal folate; endometrial thickness (5.7–12 mm) fit for IUI	—	[140]
Human study	Human clinical trials, MIRACLE trial (randomized, double-blind, placebo-controlled); green tea extract (GTE, contains EGCG) human studies	Human, GTE (150 mg EGCG twice daily, total 300 mg EGCG/d, decaffeinated) for 3 y; 4-wk run-in period to assess tolerance	Human safety, well-tolerated; only grade 1/2 ALT/AST elevation (EGCG group 3.2% vs. placebo 1.2%), no grade 3/4 liver injury; no discontinuation due to liver enzyme elevation; no direct liver dysfunction or pathological damage	—	[141]

Abbreviations: AE, adverse event; AKT, protein kinase B; ALP, alkaline phosphatase; ALT, alanine aminotransferase; AMPK, AMP-activated protein kinase; AST, aspartate aminotransferase; BPA, bisphenol A; CD69, cluster of differentiation 69; Col1a1, collagen type I alpha 1 chain; Col3a1, collagen type III alpha 1 chain; CYP4A14, cytochrome P450 family 4 subfamily A member 14; Cyp7a1, cytochrome P450 family 7 subfamily A member 1; EGCG, epigallocatechin gallate; Elovl6, ELOVL fatty acid elongase 6; FADS2, fatty acid desaturase 2; GTE, green tea extract; HFD, high-fat diet; HIF-1α, hypoxia-inducible factor 1-alpha; IUI, intrauterine insemination; LDH, lactate dehydrogenase; Lss, lanosterol synthase; NAFLD, nonalcoholic fatty liver disease; NASH, nonalcoholic steatohepatitis; NF-κB, nuclear factor kappa B; Nrf2, nuclear factor erythroid 2–related factor 2; p21, cyclin-dependent kinase inhibitor 1; p53, tumor protein p53; SCD1, stearoyl-CoA desaturase 1; SCFAs, short-chain fatty acids; SIRT3, sirtuin 3; SIRT5, sirtuin 5; SIRT6, sirtuin 6; SREBP1, sterol regulatory element-binding protein 1; α-SMA, alpha-smooth muscle actin; TLR4, Toll-like receptor 4; TNF-α, tumor necrosis factor alpha; TJPs, tight junction proteins; ZO-1, Zonula occludens-1.

#### Regulation of hepatic inflammatory response by EGCG–gut microbiota

In addition to excessive accumulation of hepatic lipids, disturbances in the intestinal environment have the potential to lead to a sustained inflammatory response in the liver, further contributing to liver injury, liver fibrosis, and cirrhosis [142]. Both lipotoxicity induced by excess fatty acids in the liver and translocation of gut endotoxins activate TLR4 signaling, which further induces the NF-κB pathway, causing persistent inflammation [86]. Additionally, gut microbiota can trigger hepatic inflammation by interfering with the TLR4/MyD88/NF-κB pathway, a key proinflammatory signaling cascade that promotes the expression of cytokines such as TNF-α, IL-6, and IL-1β, thereby driving liver injury and inflammation [143]. As shown in Figure 3, dietary supplementation with EGCG is proven to inhibit the TLR4/MyD88 pathway, alleviating inflammation through its antimicrobial, antioxidant and prebiotic properties [144]. And the remission process can be attributed in part to the interaction of EGCG with the gut microbiota shown in Table 2. Studies found a negative correlation between the relative abundance of beneficial gut microbiota such as *Akkermansia* and *Faecalibaculum* and the levels of inflammatory factors in the host [143]. EGCG facilitates the colonization of beneficial probiotics such as *Akkermansia* , *Dubosiella*, *Lactobacillus* and *Bifidobacterium*, whereas reducing the invasion of harmful bacteria from the *Bacteroidetes* and *Desulfovibrio* [145]. This regulation of the intestinal microbiota reduces the level of LPS, inhibits the expression of proinflammatory cytokines such as TNF-α, IL-1β and IL-6 and subsequently alleviates liver inflammation through the TLR4/MyD88/NF-κB signaling pathway [78,87,138]. Furthermore, EGCG alleviates liver fibrosis by reducing intracellular calcium levels and downregulating phospholipase C epsilon 1 (PLCE1) expression. PLCE1 is a phosphoinositide-specific phospholipase C that converts phosphatidylinositol-4,5-bisphosphate into inositol-1,4,5-trisphosphate and diacylglycerol, thereby triggering Ca^2+^ release and profibrotic responses in activated hepatic stellate cells (aHSCs), which downregulation diminishes inflammation associated with liver fibrosis [146]. By inhibiting the nuclear translocation of transcription factor EB, a master transcriptional regulator of the lysosomal and autophagy pathways, EGCG not only suppresses aHSC activation and lipid autophagy but also potentially modulates inflammatory pathways, reducing liver inflammation and slowing fibrosis progression [146].

### EGCG–gut microbiota–brain axis

With the rapid development of microbiomics, numerous studies have found significant modulation of the neuroimmune system in patients with nonspecific inflammatory bowel disease and functional dyspepsia [147,148]. Peripheral nerves are able to transmit the immune status of the gut to the central nervous system (CNS), which then issues commands to regulate the gut microbiota [149]. As a result, bidirectional regulation of the brain–gut axis is beginning to be noticed in pathophysiology. Research in neurological disorders such as anxiety, autism, depression, Alzheimer's disease, Parkinson's disease, etc., has also expanded from lesion intervention in the brain to targeting the gut microbiota [[150], [151], [152], [153]]. EGCG has been proven to prevent or alleviate diseases associated with disorders caused by CNS disorders [154,155]. Its inhibitory effect on neuronal apoptosis, repair of damaged neurons, and amelioration of inflammation have been demonstrated in rat, drosophila, and zebrafish models [156,157]. However, in human body, EGCG usually enters the gut first as a dietary intake before acting elsewhere [158], and in an in vitro blood-brain barrier model composed of human brain endothelial cells, pericytes, and astrocytes, EGCG's ability to directly cross the intact blood-brain barrier is limited, with low permeability (2.8% ± 0.1% in 30 min) [159]. So, it is more common to study the synergistic modulatory effects of EGCG and gut microbiota on the CNS.

#### Regulation of hypothalamic-pituitary-adrenal (HPA) axis by EGCG–gut microbiota

The HPA axis serves as a stress circuit, a mediating pathway for psychosocial stress, environmental stress, and other induced inflammatory and stress responses in recipients [22]. Stress stimulates the hypothalamus, prompting it to release corticotropin-releasing hormone (CRH) [160]. CRH acts on the pituitary gland, prompting it to release adrenocorticotropic hormone (ACTH) into the bloodstream. ACTH binds to the melanocortin 2 receptor to stimulate the secretion of corticotropin (CORT), which raises blood glucose levels and suppresses the immune function of the body. In addition, CORT can negatively feedback regulate CRH and affect CRH synthesis. If feedback signals are retarded or the individuals is chronically stressed, allowing these acute stress processes to gradually become chronic and ongoing physiological states, there may be detrimental effects on the neuropathology network nodes. Cumulative neuropathology will lead to impaired cognitive-emotional processing, decrease responsiveness, and positive feedback to a state of chronic stress or inflammation. Research in mouse models of aging has demonstrated that EGCG can modulate gut–brain communication by increasing levels of the adrenal hormone dehydroepiandrosterone (DHEA), a functional component of the HPA axis [161], and by attenuating HPA axis hyperactivity (as evidenced by reduced CRH, ACTH, and corticosterone levels) whereas simultaneously increasing the levels of SCFAs in the host [25]. Feng et al. [162] found that EGCG promotes the body's ability to regulate CORT levels, which they linked to an observed increase in plasma DHEA levels, a steroid hormone associated with the HPA axis. Lee et al. [163] demonstrated that intraperitoneal EGCG (25 mg/kg) significantly reduced synthase-stimulated elevations in plasma CORT, CRH, and ACTH in a post-traumatic stress disorder rat model, proposing that this suppression of HPA axis hyperactivity involves attenuating neuroinflammation and reversing stress-induced neurosteroid (allopregnanolone and progesterone) alterations. Luo et al. [25] found that EGCG inhibited the activation of the HPA axis and significantly reduced CRH, ACTH, and corticosterone, which may be related to EGCG's ability to optimize the abundance of gut microbiota and improve gut barrier integrity.

#### Regulation of 5-HT signaling system by EGCG–gut microbiota

5-HT is a monoamine neurotransmitter widely distributed in the CNS and intestines, and plays an important role in regulating the brain’s cognition, mood, and memory [164]. 5-HT in the gut is synthesized by enterochromaffin cells (ECs) [22,147] and its receptors in turn exist in various parts of the body, possessing different regulatory roles and signaling pathways. 5-HT4 receptors in the hippocampus have been shown to play an important role in improving learning ability, alleviating cognitive impairment, and enhancing dependent memory [165]. It can be inferred from the above that the gut is able to affect parts of the host brain via 5-HT acting on the brain–gut axis. The influence of the gut is important for the regulation of 5-HT in the body. In the study of Li et al. [166], 5-HT levels decreased in the colon and increased in the hippocampus after EGCG treatment in chronic unpredictable mild-exposed rats, suggesting that EGCG may exert its role in improving depressive syndrome by mediating the homeostasis of 5-HT levels.

In addition to the ability of EGCG to regulate 5-HT metabolism and influence 5-HT receptors, recent studies have revealed that some gut microbes are promastigotes of the 5-HT precursor substance tryptophan and that their metabolite SCFAs activate endogenous 5-HT synthesis in the brain [167,168]. This suggests that EGCG may interact synergistically with the gut microbiota to modulate the brain–gut axis via 5-HT. EGCG–gut microbiota may act by influencing the regulation of the synthesis of the 5-HT substrate tryptophan. Muraleedharan et al. [22] indicated that EGCG enhance the abundance of beneficial bacteria like Akkermansia and Dubosiella, which are involved in serotonin production improving gut barrier function and increasing the synthesis of serotonin by promoting the expression of tryptophan hydroxylase (TPH1) in the gut. Similarly, Zhou et al. found that EGCG was able to elevate the levels of tryptophan-producing associated bacteria in the gut. Additionally, the EGCG–gut microbiota may act by increasing the content of SCFAs. Dalile et al. [169] found that 5-HT secretion was associated with the content of SCFAs, which may related to the ability of EGCG to repair the gut barrier [170]. For example, Li et al. [166] experimentally found a correlation between the rebound in 5-HT levels and a decrease in the tightness of the colonic tissue and the levels of inflammatory factors.

#### Regulation of neuroinflammation by EGCG–gut microbiota

Neuroinflammation is one of the important causative factors in neurodegenerative diseases [171]. Inflammatory factors can affect the brain by linking the HPA axis and affecting 5-HT levels [[172], [173], [174]]. They form a positive feedback loop with the HPA axis, leading to increased cortisol levels, whose activity in turn promotes CNS inflammation [175]. This leads to a state of chronic stress in the brain, resulting in inflammation and deterioration of important neural networks in the brain [176].

The CNS and the enteric nervous system (ENS) are interconnected. Rao et al. [177] concluded that due to their structural similarities, pathogenic factors causing CNS disease may also lead to ENS dysfunction. Excess inflammatory factors increase the excitability of intrinsic primary afferent neurons, altering sensory and motor nerve signals and causing gastrointestinal symptoms like stomach cramps or diarrhea [178]. Gut inflammation may also affect intestinal reflexes by impacting various effector tissues in the EC or ENS innervation, further influencing the CNS [179]. For example, excess inflammatory factors can trigger inflammation driven by immune T cells that mediates dopaminergic neurodegeneration.

EGCG reduces neuroinflammation by inhibiting inflammatory pathways [180], and itself and its metabolites often exert anti-inflammatory effects by acting on the MAPK/NF-κB pathway or the TLR4/MyD88/NF-κB signaling cascade. These pathways are critical regulators of neuroinflammation, controlling the activation of microglia and astrocytes, and the subsequent production of proinflammatory cytokines such as TNF-α, IL-1β, and IL-6 in the brain [180]. Yang et al. [77] found that EGCG can reshape the gut microbiota by increasing the abundance of beneficial bacteria such as Faecalibaculum and Akkermansia, while reducing pathogenic *Tuzzerella*, which improves intestinal barrier function, reduce peripheral inflammation, inhibit the TLR4/MyD88/NF-κB pathway, and ultimately relieve neuroinflammation and anxiety-like behaviors.

Gut bacteria and their mediators can influence the behavior of T cells and thus affect neuroinflammation [148,181]. EGCG modulates the gut microbiota, promoting the production of SCFAs, which play a crucial role in maintaining gut health and influencing brain function. By increasing SCFA production and enhancing the abundance of beneficial bacteria like *Akkermansia*, EGCG helps reduce inflammation and supports the integrity of the colonic barrier [22]. SCFAs are also able to activate receptors such as GPR109a on the basis of crossing the blood-brain barrier and participate in the regulation of neuroplasticity and the immune system [169]. With EGCG–gut microbiota as both, it is not difficult to predict its preventive and interventional role in neuroinflammation.

### EGCG–gut microbiota–renal axis

The kidney is an important detoxification organ in human body, with many body toxins originating from dietary intake and microbial metabolism. For example, protein fermentation metabolites and trimethylamine-N-oxides that choline and L-carnitine are converted to after metabolism by the gut and liver have toxic effects, and their overaccumulation may easily cause renal failure, resulting in progressive renal disease (chronic kidney disease) [182]. The gut microbiota interacts with the kidneys via the renal–gut axis. Paul et al. [183] found that patients with kidney disease often have a compromised gut barrier and higher gut permeability and the abundance of the gut microbiota also changes. In patients with uremia, levels of anaerobic bacteria such as *Bifidobacteria* and *K. pneumonia*e are significantly lower, and levels of *Clostridium* are significantly higher [184].

Reciprocally, alterations in the gut microbiota composition play a crucial role in the progression of renal disease [185]. For example, it was found that patients with diabetes could be accurately distinguished from diabetic nephropathic patients by variables in 2 genera (*g_Escherichia-Shigella* and *g_Prevotella_9*). In experiments with nephrectomized mice, excess *Bacteroides* and decreased levels of *Lactobacillu*s were found to be one of the hallmarks of renal failure [186]. Moreover, fecal microbiota transplantation has emerged as an effective therapeutic strategy. Multiple studies have demonstrated that transplanting healthy fecal bacteria can significantly improve renal disease-associated symptoms [[187], [188], [189], [190]].

In diabetic nephropathy, the renin–angiotensin system (RAS) is one of the centers of reciprocal mediation between the kidney and the gut microbiota [191]. Existing studies have found that EGCG oxidation-derived polymers can significantly increase the expression of angiotensinogen [192]. Renin is stored in small afferent arterioles of the renal juxtaglomerular apparatus, to which Olfr78 is also distributed. Olfr78 mediates the release of renin when stimulated by SCFAs [193,194]. In addition, angiotensin-converting enzyme (ACE) is abundantly expressed in the endothelial cells of the renal vasculature in rats and in the brush border membranes of the proximal tubules in human kidneys [195,196]. Angiotensin produced by the RAS is converted to angiotensin II by ACE, which can lead to vascular dysfunction and renal injury [197]. Zhong et al. [185] found that differences in the etiology and severity of renal disease and the animal models used in the experiments caused different changes in the production of SCFAs. SCFAs and their derivatives are able to attenuate renin expression and thus ameliorate nephropathy to some extent [198].

EGCG which has properties that mitigate metal toxicity can reduce the symptoms of nephrotoxicity by directly intervening in oxidative stress pathways, renal inflammatory pathways [62]. Yun et al. [23] administered EGCG to a hyperuricemia mouse model and found that EGCG could regulate the diversity and composition of the gut microbiota, increasing the abundance of *Faecalibacterium* and *Bifidobacterium*, whereas reducing the relative abundance of Lactobacillus. Xu et al. [199] discovered that EGCG improved the liver and kidney toxicity caused by Cisplatin by regulating the intestinal microbiota and targeting the Nrf2/Keap1 signaling axis, a master regulator of the cellular antioxidant response that, when activated by EGCG, enhances the expression of cytoprotective genes to counteract cisplatin-induced oxidative stress and organ damage. In addition, EGCG significantly enhanced the synthesis of intestinal metabolites, such as butyrate and PGE2, which then influenced the renal urate excretion transporter genes (Oat1 and Oct1) through the gut–kidney axis, thereby promoting urate excretion and lowering serum uric acid levels [23]. Furthermore, studies have shown that oral administration of EGCG alters the transcriptional levels of cytochrome P450 metabolic enzymes and transporters, including inducing the transcriptional levels of CYP3A11, CYP2C37, and CYP3A11 in the small intestine, and affects the activity and transcriptional levels of organic anion transporters ingested by the kidneys [200]. Although the synergistic intervention of EGCG–gut microbiota in nephropathy has not been proved by too many experiments with limited experimental data. However, it was inferred from polyphenolic experiments that EGCG–gut microbiota can exert synergistic ameliorative effects on nephropathy through the renal–gut axis. Hua et al. [201] found that the pomegranate rind polyphenol Punicalagin enriches SCFA-producing gut bacteria such as *Akkermansia* and *Lachnospiraceae*, restores the intestinal barrier, and attenuates renal inflammation via the gut–kidney axis to effectively modulate HFD-induced diabetic kidney injury. Li et al. [202] found that direct treatment with SCFAs also provided a protective effect on the kidneys by increasing the level of gut microbiota producing *Prevotella* and *Bifidobacterium*. As a bioactive polyphenol like Punicalagin, EGCG shares core mechanisms such as regulating gut microbiota-SCFA crosstalk [23], repairing intestinal barrier [87], and suppressing renal inflammation [62]. Thus, we infer EGCG likely exerts synergistic nephroprotective effects via an analogous gut–kidney axis.

### EGCG–gut microbiota–lung axis

EGCG's antioxidant and anti-inflammatory properties have been extensively explored in lung disease research [[203], [204], [205], [206]]. The gut microbiota is essential for respiratory health, as dysbiosis has been closely linked to various pulmonary diseases such as asthma, chronic obstructive pulmonary disease (COPD), and lung infections [207]. In the gut–lung axis, microbial metabolites like SCFAs modulate immune responses and influence lung inflammation. EGCG exerts antioxidant effects in the gut, reducing oxidative stress and inhibiting inflammation pathways like NF-κB and NLRP3 inflammasome activation. It can also regulate the gut microbiota by promoting the growth of beneficial bacteria (*Bifidobacterium* and *Lactobacillus*), enhancing gut barrier function, and inhibiting harmful bacteria (*Escherichia coli* and *K. pneumoniae*), thereby indirectly reducing lung inflammation [207]. Xu et al. [208] demonstrated that tea polyphenols such as EGCG can prevent or alleviate COVID-19 by inhibiting microbiota dysbiosis. Li et al. [209] found EGCG can reverse the decrease in the abundance of *Clostridia* and the increase in the abundance of *Deltaproteobacteria* and *Epsilonproteobacteria* caused by obesity, thereby alleviating the progression of lung cancer. Similar polyphenols, such as resveratrol and Rosa roxburghii Tratt polyphenol, have also been shown to improve lung health through the gut–lung axis [210,211]. For example, resveratrol can reduce gut inflammation, alleviating asthma and COPD symptoms [210]. EGCG likely employs comparable mechanisms, including reducing inflammatory mediators (IL-1β, TNF-α) [212] and modulating the gut microbiota [209]. Furthermore, Cohen et al. [206] found that in biopsy samples of patients with lung disease who took EGCG orally, multiple proinflammatory and stress pathways were downregulated, and the signaling pathways related to idiopathic pulmonary fibrosis were inhibited, verifying the potential of EGCG to alleviate lung diseases through the gut–lung axis. Thus, EGCG may alleviate lung diseases by regulating the gut microbiota, strengthening the gut barrier, and suppressing inflammation in both the gut and lungs.

### Other EGCG–gut microbiota axes

In addition to the axes mentioned above, proposed axes such as EGCG–gut–heart, EGCG–gut–skin, EGCG–gut–bone, and EGCG–gut–reproductive have emerged. Although direct evidence linking EGCG to these axes remains lacking, existing studies suggest that EGCG can ameliorate pathological conditions in the corresponding organs. Given the critical role of gut microbiota in the pathogenesis of these organ-related diseases, it is hypothesized that EGCG may exert beneficial effects on lung, skin, bone, and reproductive system diseases via the gut–organ axes.

A common condition of functional impairment of the heart, which primarily pumps oxygen and blood to the host, is heart failure (HF). In some cases, it can lead to arrhythmias, myocarditis and myocardial infarction [213]. Historically, in the realm of HF research, the neurohormonal axis was considered the principal pathway mediating HF, but experiments have found that blocking neurohormones alone does not completely stop disease progression in patients with HF [214]. Currently, with the study of the gut microbiota, the ecological dysregulation of the gut microbiota has been experimentally identified as a potential player in the pathogenesis and further development of HF, which often manifests itself in the form of reduction in microbial diversity and alterations in the ratio of key microbial species. In turn, persistent chronic inflammation and higher endotoxin levels, mediated by a disturbed gut microbiota, will further affect the extent of HF [215]. Pasini et al. [216] found that patients with HF may have an overrepresentation of both pathogenic bacteria such as *Campylobacter*, *Shigella*, *Salmonella*, *Yersinia enterocolitica* and *Candida* growing in the gut. Luedde et al. [217] noted a significant decrease in the proportions of *Coriobacteraceae, Erysipelotrichaceae*, and *Ruminalococcaceae* in the gut of patients with HF. Although direct experimental evidence for the synergistic regulation of the heart–gut axis by the EGCG–gut microbiota complex is currently limited, studies have demonstrated the beneficial effects of EGCG in cardiac-related diseases. These effects are often attributed to its direct actions, such as exerting antioxidant capacity, reducing myocardial cell apoptosis, targeting increased expression of miR-450b-5p, and inhibiting LPS and endotoxin [[218], [219], [220], [221]]. It has been fully confirmed in similar polyphenol quercetin, which is able to mitigate mitochondrial autophagy in the heart with its antioxidant capacity, inhibit the activation of the NF-κB pathway to reduce inflammation, and increase the α and β diversity of the gut microbiota to mitigate the imbalance of the intestinal environment induced by cardiac diseases, and also stabilize the ecological balance of the intestinal tract to slow down the onset of cardiac diseases [222,223]. Additionally, oral catechins have been clinically proven to correct diastolic dysfunction in children with cardiomyopathy [218], which further indicates that it is reasonable to postulate that EGCG, a polyphenol with high bioactivity like quercetin, can also synergize with the gut microbiota in the regulation of the heart.

Dietary polyphenols have been shown to have beneficial therapeutic effects on various skin diseases [[224], [225], [226]]. EGCG has demonstrated significant potential in alleviating skin inflammation and improving skin health through its antioxidant and anti-inflammatory properties. For example, it has been demonstrated to increase the minimal erythema dose and reduce the harmful effects of UV exposure on skin [227,228], and modulates the expression of COX-2 and p38 MAPK pathways, mitigating UVB-induced oxidative stress and inflammation [229]. The gut microbiota, via the gut–skin axis, impacts skin health, such as dysbiosis is associated with acne and psoriasis [230]. EGCG promotes the growth of beneficial gut bacteria such as *Lactobacillus* and *Bifidobacterium* [78] and inhibiting harmful bacteria [145], enhancing gut barrier function and reducing systemic inflammation [231]. This dual mechanism highlights EGCG's potential as an effective dietary intervention for skin health.

EGCG has been shown to have beneficial effects on bone-related diseases. It alleviates arthritis by modulating Nrf2, HO-1, and cytokine levels [232] and promotes osteoblast differentiation to alleviate osteoporosis [233]. Additionally, tea polyphenols including EGCG improve bone health via the gut–bone axis. Specifically, in retinoic-acid osteoporotic mice, they can restructured the intestinal microbiota by increasing *Firmicutes* and *Lactobacillus* whereas decreasing *Bacteroidetes* and *Bacteroides*, and repair the damaged intestinal barrier by restoring goblet-cell density and upregulating tight junction proteins ZO-1 and Claudin-1, and consequently increased trabecular bone density and decreased osteoclast density [234]. In the research of Han et al. [235], it was found that EGCG can increase the abundance of beneficial bacterial communities, including *Prevotellaceae* and *Ruminococcus*, and inhibit the growth of pathogenic bacteria such as *Peptostreptococcaceae* to alleviate osteoporosis. Notably, *Peptostreptococcaceae* was negatively correlated with bone mineral density and bone volume fraction, and positively correlated with trabecular separation; suppressing these detrimental bacteria helps restore microbial balance and protect bone health [235]. EGCG may alleviate bone-related diseases through the gut–bone axis by improving gut microbiota composition [234, 235].

EGCG can relieve reproductive related diseases [236], including infertility [237], gynecological diseases [238], testicular injury [239]. Its protective effects are primarily attributed to potent antioxidant and anti-inflammatory activities, which mitigate oxidative stress and hormonal dysregulation [[237], [238], [239]]. For female reproductive diseases, Zhang et al. [237] found that EGCG acts mainly by regulating ROS (reactive oxygen species) levels, which affect the expression of CAT, superoxide dismutase 1 (SOD1), superoxide dismutase 2 (SOD2) and glutathione peroxidase, which delays germ cell and oocyte infertility. Chen et al. [240] found that EGCG can activate NRF2 to reduce oxidative DNA damage, inhibit overactivation of primary follicles and follicular atresia, and independently alleviate cyclophosphamide induced ovarian damage. For male related diseases, Fang et al. [241] found that fibroin microneedles containing EGCG mitigated atrazine-induced testicular toxicity. Gu et al. [242] discovered that EGCG prevents precocious puberty caused by obesity by regulating the intestinal flora and altering metabolic pathways, particularly tryptophan metabolism, as revealed by fecal metabolomics. Hassan et al. [239] demonstrated that EGCG improves testicular toxicity and increases the activity of antioxidant enzymes by inhibiting oxidative stress. EGCG can improve lead (Pb)-induced testicular injury by increasing Cyp19 gene expression and serum E2 level. Intestinal microbiota plays a regulatory role in both male and female reproduction. For females, it exerts various influences on female reproduction by regulating hormone regulation, immune function, nutrient metabolism, inflammatory pathway and reproductive tract ecosystem, whereas for males, it regulates male hormones, insulin sensitivity, immune system and testicular microbiota [243]. Because EGCG has a good ability to regulate intestinal flora, it is speculated that EGCG exerts an alleviating effect on reproductive diseases through the entero-reproductive axis. Despite the lack of sufficient direct evidence linking EGCG to the alleviation of diseases through the gut–lung axis, gut–skin axis, gut–bone axis, and gut–reproductive axis, existing indirect evidence allows for speculation on the underlying mechanism of EGCG–gut–organ axis.

### Human clinical evidence on EGCG and systemic health

The exploration of EGCG in human trials has primarily focused on its systemic health benefits, particularly in metabolic regulation, neuroprotection, and cancer prevention. Although direct evidence linking EGCG to gut health in clinical settings remains limited, emerging in vitro and preclinical studies suggest that the gut microbiome may serve as a critical mediator of its therapeutic effects [10,35,221].

Colorectal cancer prevention trials, such as the Minimizing the Risk of Metachronous Adenomas of the Colorectum with Green Tea Extract (MIRACLE) randomized controlled trial, demonstrate that EGCG (300 mg/d) shows modest gender-specific benefits in reducing adenoma recurrence, particularly in men [103,104]. A meta-analysis of 5 RCTs further supports EGCG’s preventive potential against colorectal cancer recurrence [104]. Mechanistically, EGCG may act via apoptosis induction, cell cycle arrest, and modulation of DNA methylation, though its effects on gut microbiota remain unproven [103]. Notably, recent in the vitro fermentation experiment suggest that EGCG’s health benefits may be partially mediated by its ability to reshape gut microbial communities (promoting beneficial taxa like Bacteroides and Christensenellaceae whereas suppressing pathobionts such as Fusobacterium varium) [10,102].

For metabolic health, a randomized crossover study in healthy women, 752–800 mg EGCG in a single-dose delayed gastric emptying and improved postprandial lipid profiles (reduced TG, attenuated HDL decline) [244,245]. In type 2 diabetes, a trial in patients (both men and women aged 40–70 y) found that 300 mg/d for 2 mo lowered BMI and blood pressure but only in carriers of the FTO-rs9939609 A allele [246]. These genotype-dependent outcomes may reflect underlying differences in gut microbiota composition, as pilot trials with oolong tea (rich in EGCG derivatives) showed stronger α-diversity improvements in overweight individuals (BMI > 23.9) compared with lean participants [247]. The observed correlation between baseline microbial imbalance and EGCG responsiveness highlights the need for future studies to incorporate microbiome profiling in metabolic intervention trials.

Additionally, EGCG shows promise in neurological and cognitive disorders. In rib fracture patients, 100 mg EGCG twice daily for 10 d significantly reduced pain (from 8 to 4) and improved breathing function, likely via TLR4 and nitric oxide synthase (NOS) inhibition [248]. In multiple sclerosis, 600–1200 mg/d EGCG for 4 mo improved motor performance. Similarly, in cognitive domains, EGCG appears most effective when combined with behavioral interventions [249]. In fragile X syndrome, 5–7 mg/kg/d EGCG plus cognitive training for 3 mo significantly improved visual memory and adaptive function, with sustained benefits posttreatment [250]. In apolipoprotein E ε4 (APOE-ε4) carriers with subjective cognitive decline, in older adults showed that adding EGCG to a 12-mo lifestyle program led to preliminary cognitive stabilization, though full data are pending [251]. The neuroprotective effects of EGCG may be indirectly linked to its modulation of gut-derived metabolites. For example, 4-phenylbutyrate, a major EGCG metabolite, was strongly associated with specific microbial taxa in fermentation experiments [10,102], suggesting that microbial transformation of EGCG could generate bioactive compounds with systemic effects.

Despite these promising results, the human clinical evidence for EGCG remains fragmented, particularly regarding its direct impact on gut health. Although in vitro and animal studies demonstrate that EGCG can modulate microbial diversity, promote SCFA production [247], and generate metabolites with anti-inflammatory properties [10], these findings have yet to be validated in large-scale human trials. Future research should prioritize longitudinal studies that integrate microbiome analyses with clinical outcomes, leveraging advances in multiomics to unravel the complex interplay between EGCG, gut microbes, and host physiology. By bridging this gap, the therapeutic potential of EGCG could be fully realized, not only as a standalone supplement but as a cornerstone of microbiome-targeted precision medicine.

## Challenges and Prospects

In summary, this review has supplied a comprehensive understanding of the structural characteristics, absorption, and metabolism of EGCG, as well as its intricate interactions with gut microbiota and the associated health implications. EGCG demonstrates significant potential for regulating gut health by enhancing the diversity of beneficial bacteria, fortifying the intestinal barrier, and optimizing nutrient metabolism. Notably, EGCG's metabolic process, which involves both enzymatic and microbial activities in the liver, small intestine, and gut, plays a crucial role in shaping its bioactivity and excretion.

The review highlights that EGCG and its metabolites can modulate gut microbiota, offering a promising approach for the prevention and management of chronic diseases such as obesity, colitis, and neurodegenerative disorders. Furthermore, the interactions between EGCG and gut microbiota extend beyond the gut, influencing systemic health through various axes, including gut–liver, gut–brain, gut–kidney, and gut–lung axes. These interactions contribute to the regulation of hepatic glucose metabolism, fatty acid homeostasis, neuroinflammation, and renal function, among others.

However, several challenges and questions remain to be addressed in future research. A more in-depth understanding of the specific mechanisms by which EGCG exerts its beneficial effects is required, particularly regarding the identification of target microbes in the gut microbiome and the bioactive metabolites produced through microbial transformation. First, the interindividual variability in EGCG metabolism and bioavailability remains a significant barrier, driven by genetic polymorphisms in key enzymes and transporter systems [27]. For example, COMT gene variants, such as the G/G genotype, are associated with higher enzymatic activity, leading to increased methylation and faster clearance of EGCG compared with individuals with the low-activity A/A or A/G genotypes, which is supported by a randomized controlled trial showing that postmenopausal women with the G/G COMT genotype exhibited significantly higher plasma EGCG concentrations after 12 mo of supplementation compared with those with the A/A or A/G genotypes [252]. Similarly, FTO-rs9939609 polymorphism (A allele carriers) has been linked to greater improvements in BMI and diastolic blood pressure after EGCG supplementation in patients with type 2 diabetes, highlighting the role of host genetics in therapeutic response [246]. These findings underscore the critical role of host genetics in EGCG’s pharmacokinetics and pharmacodynamics. Metabolic and therapeutic response variability highlights the need for personalized strategies such as genotype-guided dosing and tailored combination therapies. Future studies should prioritize multiomics analyses to dissect crosstalk between genetic polymorphisms, microbial metabolism, and EGCG bioactivity, ultimately facilitating precision nutrition for chronic disease prevention and management. Second, gut microbiota dynamics complicate EGCG’s metabolic outcomes. Interindividual variations in microbiota composition which driven by host factors such as transit time, intestinal inflammation, and BMI significantly alter metabolic pathways [[253], [254], [255]]. This microbial heterogeneity acts as a key confounder, leading to inconsistent metabolite profiles across individuals and obscuring causal relationships between microbiome targets and health outcomes [255]. Consequently, failure to control for these variables limits the identification of precise target metabolites and hinders the development of standardized dosing protocols. Third, concerns about long-term safety persist, as current evidence is primarily derived from short-term studies (≤3 mo) with limited data on high-dose effects beyond 800 mg/d [141]. Long-term high-dose EGCG also caused gut microbiota dysbiosis, including a 20% reduction in beneficial *Akkermansia* abundance and decreased α-diversity, which may disrupt gut health homeostasis and a high dose of 800 mg/d even led to mild elevation of liver enzymes such as alanine aminotransferase and aspartate aminotransferase in 5% of participants, implying hepatic metabolic burden that might indirectly affect gut–liver crosstalk [241]. Additionally, no long-term monitoring of core gut–health indicators such as intestinal barrier integrity, SCFA metabolism) has been conducted, leaving uncertainty about EGCG’s long-term impacts on intestinal mucosal immunity and gut metabolite homeostasis which gaps hinder its clinical translation for the gut–health axis [256]. Additionally, strategies for enhancing the bioavailability of EGCG and developing targeted delivery systems to improve its therapeutic potential warrant further exploration. Conducting well-designed human trials with sufficient sample sizes is also essential to establish robust evidence for the health benefits of EGCG and to guide the development of EGCG-based interventions for clinical application.

From a future therapeutic potential perspective, these gaps also point to actionable directions.

### Microbiome–EGCG interactions

Systematic studies are needed to map the full spectrum of microbial enzymes and metabolites involved in EGCG transformation. For example, synthetic biology could engineer probiotics capable of selectively producing anti-inflammatory EGCG metabolites, whereas preclinical models can identify microbial biomarkers predictive of EGCG efficacy.

### Personalized delivery systems

Innovations in nanotechnology, such as pH-sensitive nanoparticles or colon-targeted delivery systems, could enhance EGCG bioavailability by protecting it from degradation in the stomach and directing its release to the colon. These strategies may synergize with microbiota modulation to maximize therapeutic outcomes.

### Long-term safety and clinical translation

Large-scale, multicenter trials with longitudinal microbiota and metabolomic profiling are essential to establish safe dosage thresholds and monitor long-term effects on gut–liver and gut–brain axes. Regulatory frameworks for EGCG-based interventions should also incorporate microbiota stability metrics (*Akkermansia* abundance, SCFA levels) to ensure safety and efficacy.

By addressing these challenges through interdisciplinary collaboration, EGCG could evolve from a dietary supplement into a cornerstone of microbiome-targeted therapies for chronic diseases.

## Author contributions

The authors’ responsibilities were as follows – JY, WC: conceived and designed the review; JY, WC, JC: drafted the manuscript; JY: generated the figures; JZ: acquired funding and had primary responsibility for final content; and all authors: edited and revised the manuscript, read and approved the final manuscript.

## Data availability

Data described in the manuscript, codebook, and analytic code will be made available on request pending application and approval.

## Funding

This study is supported by the Fundamental Research Funds for the Zhejiang Provincial Universities under Grant (226-2024-00214); the “Pioneer” and “Leading Goose” R&D Program of Zhejiang under Grant (2023C02041); and the Research Project on Optimization of Plantation Management and Processing Technology of Matcha Tea Plantation in Liandu District.

## Conflicts of interest

The authors report no conflict of interest.
